# Asperflaloids A and B from *Aspergillus* *flavipes* DZ-3, an Endophytic Fungus of *Eucommia* *ulmoides* Oliver

**DOI:** 10.3390/molecules26123514

**Published:** 2021-06-09

**Authors:** Wan Liu, Yu Liu, Fan Yang, Shouye Han, Jia Zhang, Hui Yang, Zhongbin Cheng, Qin Li

**Affiliations:** 1 School of Pharmacy, Henan University, Kaifeng 475004, China; 18737806806@163.com (W.L.); liuyu5230710@163.com (Y.L.); Y18992588130@126.com (F.Y.); hanshouye123@163.com (S.H.); z919395@126.com (J.Z.); 2 Eucommia Ulmoides Cultivation and Utilization of Henan Engineering Laboratory, Kaifeng 475004, China

**Keywords:** *Aspergillus* *flavipes* DZ-3, asperflaloids A and B, structural identification, bioactivity

## Abstract

The fungus strain DZ-3 was isolated from twigs of the well-known medicinal plant *Eucommia ulmoides* Oliver and identified as *Aspergillus flavipes*. Two new alkaloids, named asperflaloids A and B (**1** and **2**), together with 10 known compounds (**3**–**12**) were obtained from the EtOAc extract of the strain. Interestingly, the alkaloids **1**–**4** with different frameworks are characterized by the presence of the same anthranilic acid residue. The structures were established by detailed analyses of the spectroscopic data. The absolute configuration of asperflaloids A and B was resolved by quantum chemistry calculation. All compounds were screened for their inhibitions against α-glucosidase and the antioxidant capacities. The results were that compound **3** had an IC_50_ value of 750.8 μM toward α-glucosidase, and the phenol compounds **7** and **8** exhibited potent antioxidant capacities with IC_50_ values 14.4 and 27.1 μM respectively.

## 1. Introduction

Fungi are widespread in natural environments and have conquered almost all ecological niches. Moreover, fungi produce many structurally intriguing molecules, many of which exhibit promising pharmacological properties, including the famous antibiotics penicillin and cephalosporin C [1,2]. *Aspergillus*, a genus of filamentous fungi, is noted for its medical and commercial significance. Species in *Aspergillus* have been proved to be prolific and are considered as an important source of chemical leads with hopeful biological activities [3,4]. Among the *Aspergillus* species, *Aspergillus flavipes* was an outstanding member; recent chemical research of the species resulted in the production of plenty of molecules with new structures and obvious biological activities, such as structurally complex merocytochalasans [5,6,7], lumazine peptides possessing significant antibacterial and NF-κB inhibitory activities [8], unusual chlorinated PKS-NRPS hybrid metabolites bearing potent pancreatic lipase inhibitory activity [9], and rare prenylated phenylbutyrolactones [10].

**Figure 1:**
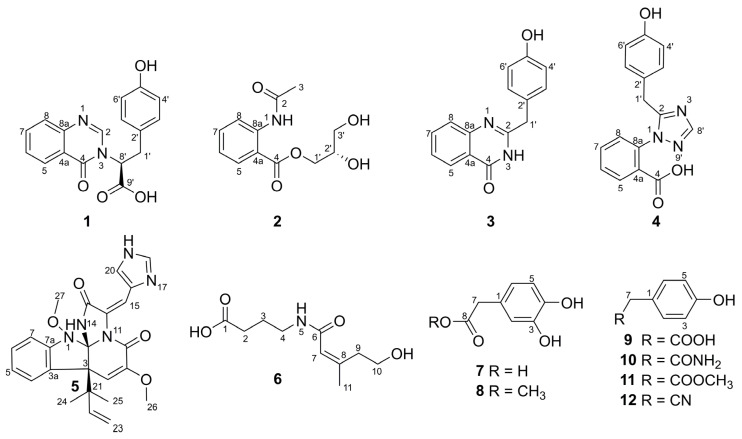
Structures of compounds **1**–**12** from *Aspergillus flavipes* DZ-*3*.

In our continuous efforts to search for bioactive natural products from fungus [11,12,13,14,15], an endophytic fungus *Aspergillus flavipes* DZ-3 was isolated from the twigs of the medicinal plant *Eucommia ulmoides* Oliver. The ^1^H-NMR spectrum and thin layer chromatography examination using the modified Dragendorff’s reagent of the EtOAc extract indicated the presence of aromatic alkaloids. Extensive chromatographic separation of the large-scale fermentation were carried out, which resulted in the isolation of 12 compounds **1**–**12** (Figure 1), including two new alkaloids. All compounds were screened for their inhibitions against α-glucosidase and the antioxidant capacities. Herein, the isolation, structure determination, and bioactivities of compounds **1**–**12** are described.

## 2. Results

**Figure 2:**
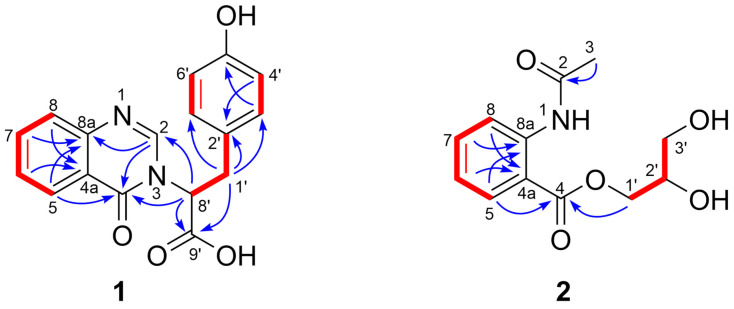
^1^H-^1^H COSY (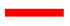) and HMBC (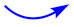) correlations of **1** and **2**.

**Figure 3:**
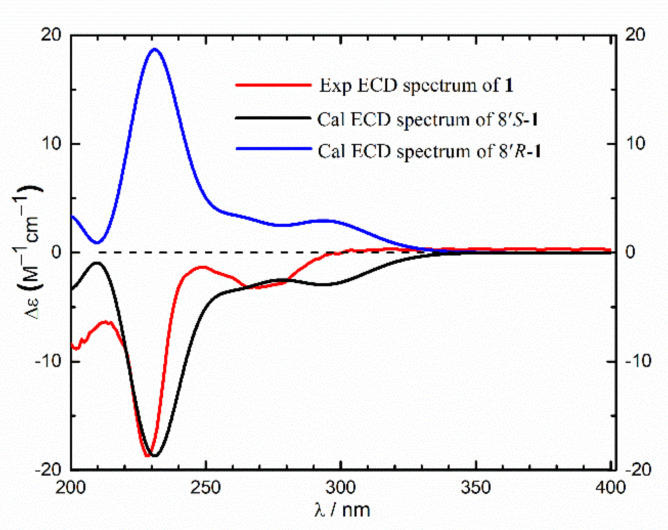
Experimental and calculated electronic circular dichroism (ECD) spectra of **1** in MeOH (σ = 0.3, UV correction = −5 nm).

Asperflaloid A (**1**) has a molecular formula of C_17_H_14_N_2_O_4_ (309.0879 [M − H]^−^, calcd for C_17_H_13_N_2_O_4_^−^, 309.0881) as determined by the HRESIMS and NMR data (Table 1, Figures S1–S6, S33), bearing 12 degrees of unsaturation. The ^1^H-NMR spectrum showed an ABCD aromatic spin system for an ortho-disubstituted benzene ring [δ_H_ 7.62 (1H, d, *J* = 8.1 Hz); 7.80 (1H, dd, *J* = 8.1, 7.5 Hz); 7.54 (1H, dd, *J* = 7.9, 7.5 Hz); 8.20 (1H, d, *J* = 7.9 Hz)]; and an AABB aromatic spin system for a para-disubstituted benzene ring [δ_H_ 6.93, (2H, d, *J* = 8.3 Hz); 6.60 (2H, d, *J* = 8.3 Hz)]. Comprehensive analyses of 2D NMR (COSY, HSQC, HMBC) data revealed that the structure of **1** consists of a quinazoline moiety and a phenylpropionic acid moiety (Figure 2). The COSY relationships connected the protons from H-5 (δ_H_ 8.20) to H-8 (δ_H_ 7.62), while the HMBC correlations between H-5 and C-4 (δ_C_ 162.3) and from H-2 (δ_H_ 7.94) to C-8a (δ_C_ 148.5) and C-4 assigned the quinazoline ring. The phenylpropionic acid moiety was established by ^1^H-^1^H COSY relationship between H_2_-1′ (δ_H_ 3.44, 3.53) and H-8′ (δ_H_ 5.40), and HMBC correlations from H_2_-1′ to C-2′ (δ_C_ 128.4), C-3′ (δ_C_ 131.1), C-7′ (δ_C_ 131.1), in association with HMBC correlation from H-8′ to the carboxylic carbon C-9′ (δ_C_ 172.2). The above two moieties were connected by a C-N bond supported by the HMBC correlations from H-8′ to C-2 (δ_C_ 148.6) and C-4. Thus, the gross structure of **1** was assigned as depicted. The absolute configuration of the only chiral center C-8′ (δ_C_ 62.3) in **1** was determined by comparisons of experimental electronic circular dichroism (ECD) with calculated ECD spectra (Figure 3, Figure S35). The calculations of the ECD spectrum of (8′*S*)-**1** and its enantiomer (8′*R*)-**1** were performed using b3lyp/6–31+g(d,p) optimized geometries after conformational searches via the MMFF94S force field at the b3lyp/6–31+g(d,p) level in methanol. The experimental ECD spectrum of **1** showed an ECD curve with Cotton effects around 228 (−) and 270 (−) nm, respectively (Figure 3). The calculated ECD spectrum for (8′*S*)-1 showed a similar ECD curve with Cotton effects at 231 nm (−) and 290 (−) nm, allowing the assignment of the 8′*S* configuration for **1**.

**Figure 4:**
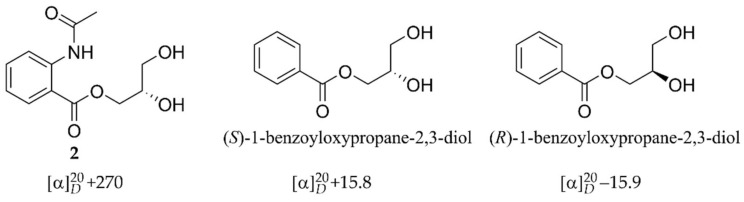
Structures and specific rotations of **2** and (*S*/*R*)-1-benzoyloxypropane-2,3-diol.

The molecular formula of compound **2** was determined to be C_12_H_15_NO_5_ (254.1024 [M + H]^+^, calcd for C_12_H_16_NO_5_^+^, 254.1023) by the HRESIMS and NMR data (Table 1, Figures S7–S12, S34), suggesting six degrees of unsaturation. The ^1^H-NMR spectrum exhibited the resonances of four aromatic protons [δ_H_ 8.11 (1H, dd, *J* = 8.1, 1.6 Hz); 7.18 (1H, dd, *J* = 8.0, 7.9, 1.0 Hz); 7.57 (1H, dd, *J* = 8.3, 7.9, 1.6 Hz); 8.45 (1H, dd, *J* = 8.3, 1.0 Hz)] for an ABCD aromatic spin system, two oxygenated methylenes [δ_H_ 4.45 (1H, dd, *J* = 11.4, 4.1 Hz), 4.34 (1H, dd, *J* = 11.4, 6.3 Hz); 3.65 (2H, d, *J* = 5.7 Hz)], an oxygenated methine [δ_H_ 3.99 (1H, m)], and a methyl [δ_H_ 2.21 (3H, s)]. The ^13^C-NMR and HSQC spectra resolved six aromatic carbons for a benzene ring (δ_C_ 141.8, 118.1, 132.2, 124.3, 135.2, 122.1), two carbonyl carbons (δ_C_ 169.1, 171.6) for ester/amide groups, two oxygenated methylene carbons (δ_C_ 67.5, 64.0), and an oxygenated methine carbon (δ_C_ 71.0). The benzene ring and the two carbonyl groups covered all six degrees of unsaturation, suggesting that no additional ring exists in the structure. The ^1^H-^1^H COSY correlations from H-5 (δ_H_ 8.11) to H-8 supported the presence of an *ortho*-disubstituted benzene ring. The COSY relationships from H_2_-1′ (δ_H_ 4.45, 4.34) to H_2_-3′ (δ_H_ 3.65) via H-2′ combined with the HMBC correlation from H-1′ and H-5 to C-4 located a 2,3-dihydroxypropyl formate moiety at C-4a. The substituent group at C-8a was established to be an acetyl amino group by the HMBC correlation from H-5 to C-4 in association with the HRESIMS data, which was also supported by the chemical shifts of C-8a. The gross structure of **2** was thus determined as shown in Figure 1. In order to resolve the absolute configuration of the only chiral center C-2′ in **2**, theoretical specific rotations of the model molecules *S*/*R*-**2** were calculated at the b3lyp/6-31+g(d) level using methanol as solvent. The results were that the theoretical specific rotation of *S*-**2** (*S*-**2**: [α${]}_{D}^{20}$ +147; *R*-**2**: [α${]}_{D}^{20}$ −147) had the same sign as the experimental data of **2** ([α${]}_{D}^{20}$ +270), indicating a 2′*S*-configuration for **2**. A comparison of the specific rotations of **2**, (*S*)-1-benzoyloxypropane-2,3-diol [α${]}_{D}^{20}$ +15.8), and (*R*)-1-benzoyloxypropane-2,3-diol ([α${]}_{D}^{20}$ −15.9) supported the 2′*S*-configuration of **2** (Figure 4) [16]. Compound **2** was given the trivial name asperflaloid B.

The remaining compounds were identical to 2-(4-hydroxybenzyl)quinazolin-4(3*H*)-one (**3**) [17], penipanoid A (**4**) [18], oxaline (**5**) [19], fuscoatramide (**6**) [20], 3,4-dihydroxybenzeneacetic acid (**7**) [21], 3,4-dihydroxyphenylacetic acid methyl ester (**8**) [21], phenylacetic acid (**9**) [22], 4-hydroxybenzeneacetamide (**10**) [23], 4-hydroxy phenylacetic acid methyl ester (**11**) [24], and 4-hydroxyphenylacetonitrile (**12**) [23] based on comparisons of their NMR data (Figures S13–S32) and specific rotations with those reported in the literature.

All compounds were tested for their α-glucosidase inhibitory activities and antioxidant capacities (Table 2). We found that compound **3** inhibited α-glucosidase with an IC_50_ value of 750.8 μM and was more active than the positive control acarbose (1.33 mM). Compounds **7** and **8** exhibited strong antioxidant capacities with IC_50_ values 14.4 and 27.1 μM, respectively, which were comparable to that of vitamin C (26.7 μM). Compound **10** exhibited weak antioxidant capacity with an IC_50_ value 339.3 μM. Other compounds exhibited negligible activity at the concentration of 1 mM.

## 3. Materials and Methods

### 3.1. General Experimental Procedure

Specific rotations were measured by an SGW-1 automatic polarimeter (Shanghai Jing Ke Industrial Co., Ltd., Shanghai, China). ECD spectra were measured on an Aviv Model 420SF spectropolarimeter (Aviv Biomedical Inc., Lakewood, NJ, USA). The NMR spectra were recorded on a Bruker Avance III HD-400 NMR spectrometer. HRESIMS spectra were obtained on a Waters Xevo G2 Q-TOF spectrometer fitted with an ESI source. Semi-preparative high-performance liquid chromatography (HPLC) was undertaken on a Shimadzu LC-6AD pump (Shimadzu Co., Kyoto, Japan) using a UV detector, and a YMC-Pack ODS-A HPLC column (semipreparative, 250 × 10 mm, S-5 μm, 12 nm, YMC Co., Ltd., Kyoto, Japan) was used for separation.

### 3.2. Fungal Strain and Identification

Fungus DZ-3 was isolated from branches of *Eucommia ulmoides* Oliver. The strain was identified as *Aspergillus flavipes* based on microscopic examination and by internal transcribed spacer (ITS) sequencing. The ITS sequence has been deposited in GenBank (http://www.ncbi.nlm.nih.gov, accessed on 13 May 2021) with the accession number MZ148624. The strain DZ-3 (henuyxydz-3) was deposited at the School of pharmacy, Henan University.

### 3.3. Fermentation, Extraction, and Isolation

The fermentation was carried out in 30 Fernbach flasks (500 mL), each containing 70 g of rice. Distilled water (90 mL) was added to each flask, and the contents were soaked for 3 h before autoclaving at 15 psi for 30 min. After cooling to room temperature, each flask was inoculated with 3.0 mL of the spore inoculum and incubated at room temperature for 30 days. The fermented material was extracted successively with EtOAc (3 × 4000 mL). After evaporation under vacuum, the EtOAc extract (1.6 g) was subjected to a middle chromatogram isolated gel (MCI) with MeOH/H_2_O (10:90 → 100:0) as eluent to obtain 7 fractions (F1 to F7). Fraction F2 was further chromatographed over ODS silica gel CC eluted with MeOH/H_2_O (30:70 → 100:0) to afford ten subfractions (SF2a–SF2j). SF2a was separated on a semipreparative reversed-phase (RP) HPLC column using MeCN/H_2_O = 20:80 (3 mL/min) to give **6** (t_R_ = 7.0 min, 4.7 mg). SF2c was purified by HPLC using MeCN/H_2_O = 21:79 (3 mL/min) as eluent to give **2** (t_R_ = 14.5 min, 1.5 mg). SF2e was subjected by HPLC using MeCN/H_2_O = 34:66 (3 mL/min) to give **4** (t_R_ = 10.3 min, 4.3 mg). SF2h was purified by HPLC using MeCN/H_2_O = 50.5:49.5 (3 mL/min) as a mobile phase to give **5** (t_R_ = 6.2 min, 9.8 mg). Fraction F3 was further chromatographed over ODS silica gel CC eluted with MeOH/H_2_O (30:70 → 90:10) to afford five subfractions (SF3a–SF3e). SF3a was separated on HPLC using MeCN/H_2_O = 14:86 (3 mL/min) as eluent to afford **7** (t_R_ = 10.7 min, 12.0 mg), **8** (t_R_ = 31.5 min, 6.5 mg), **9** (t_R_ = 17.6 min, 12.9 mg), **10** (t_R_ = 7.7 min, 16.1 mg) and **12** (t_R_ = 32.9 min, 3.8 mg). SF3b was further purified on HPLC using MeCN/H_2_O = 20:80 (3 mL/min) as a mobile phase to obtain **1** (t_R_ = 40.5 min, 29.4 mg) and **11** (t_R_ = 34.7 min, 14.1 mg). Compound **3** was precipitated from SF3c.

Asperflaloid A (**1**): Brown oil; [α${]}_{D}^{20}$ –47 (c 0.08, MeOH); UV (MeOH) λmax 225 nm, 271 nm, 302 nm; ^1^H and ^13^C-NMR data, see Table 1; HRESIMS m/z 309.0879 [M − H]^−^ (calcd for C_17_H_13_N_2_O_4_^−^, 309.0881).

Asperflaloid B (**2**): Colorless oil; [α${]}_{D}^{20}$ +270 (c 0.035, MeOH); UV (MeOH) λmax 221 nm, 249 nm, 304 nm; ^1^H and ^13^C-NMR data, see Table 1; HRESIMS m/z 254.1024 [M + H]^+^ (calcd for C_12_H_16_NO_5_^+^, 254.1023).

### 3.4. α-Glucosidase Assay

The α-glucosidase inhibitory effect was assessed as described in our recently published paper [25].

### 3.5. Antioxidant Activity

The DPPH scavenging was assayed according to the reported method [26]. The DPPH radical scavenging test was performed in 96-well microplates. Samples (compounds **1**–**12**) were added to 180 μL (150 μmol/L) DPPH solution in EtOH at 20 μL solutions of different concentrations between 10 and 500 μM. After 30 min of light avoidance, absorbance at 517 nm using a microplate reader (Tecan Trading AG, Männedorf, Switzerland) was measured and the percentage of activity was calculated. All assays were performed in three replicates, and vitamin C was used as a positive control.

### 3.6. Computational Details

ECD calculations. Conformational analyses were carried out via random searching in the Sybyl-X 2.0 using the MMFF94S force field with an energy cutoff of 2.5 kcal/mol [27]. The results showed the three lowest energy conformers for *R*-**1.** The conformers were re-optimized using DFT at the b3lyp/6-31+g(d,p) level in methanol by the GAUSSIAN 09 program [28]. The energies, oscillator strengths, and rotational strengths (velocity) of the first 30 electronic excitations were calculated using the TDDFT methodology at the b3lyp/6-31+g(d,p) level in methanol. The ECD spectra were simulated by the overlapping Gaussian function (half the bandwidth at 1/e peak height, σ = 0.3, UV correction = −5 nm) [29]. To get the final spectra, the simulated spectra of the conformers were averaged according to the Boltzmann distribution theory and their relative Gibbs free energy (ΔG), theoretical ECD spectrum of the corresponding enantiomer *S*-**1** was obtained by inverse of the ECD spectrum of *R*-**1**, respectively. By comparing the experiment spectrum with the calculated ECD spectra, the absolute configuration of the chiral center C-8′ in **1** was resolved to be *S*.

Specific rotation calculations. Conformational analyses were carried out via random searching in the Sybyl-X 2.0 using the MMFF94S force field with an energy cutoff of 2.0 kcal/mol. The results showed the three lowest energy conformers for *R*-**2**. The conformers were re-optimized using DFT at the b3lyp/6-31+g(d,p) level in methanol by the GAUSSIAN 09 program. The specific rotations for each conformer were calculated using the TDDFT methodology at the b3lyp/6-31+g(d) level in methanol. The specific rotations obtained for the conformers were averaged according to the Boltzmann distribution theory and their relative Gibbs free energy (ΔG) to give the specific rotation of *R*-**2**, the specific rotation of *S*-**2** was theoretically determined to be the opposite value of *R*-**2**. By comparing the experiment data ([α${]}_{D}^{20}$ +270) with the calculated data (*R*-**2**: [α${]}_{D}^{20}$ −147; *S*-**2**: [α${]}_{D}^{20}$ +147), the absolute configuration of the chiral center C-2′ in **2** was resolved to be *S*.

## 4. Conclusions

Chemical investigation of the EtOAc extract of an endophytic fungus *Aspergillus flavipes* DZ-3 led to the isolation of two new alkaloids, named asperflaloids A and B (**1**–**2**), and 10 known compounds (**3**–**12**). The structures were established by extensive analyses of spectroscopic data (1D and 2D NMR, HRESIMS) and quantum chemistry calculation. Compound **3** exhibited inhibitory effect toward α-glucosidase with an IC_50_ value of 750.8 μM and was more active than the positive control acarbose (1.33 mM). Compounds **7** and **8** exhibited remarkable antioxidant capacities with IC_50_ values of 14.4 and 27.1 μM, respectively, which were comparable to that of the positive control vitamin C (26.7 μM).

## Figures and Tables

**Table 1:** ^1^H (400 MHz) and ^13^C-NMR (100 MHz) data of **1** and **2** (In methanol-*d_4_*).

No.	1	2		
	δ_H_ (*J* in Hz)	δ_C_	δ_H_ (*J* in Hz)	δ_C_
2	7.94, s	148.6		171.6
3			2.21, s	24.9
4		162.3		169.1
4a		122.7		118.1
5	8.20, d (7.9)	127.5	8.11, dd (8.0, 1.6)	132.2
6	7.54, dd (7.9, 7.5)	128.6	7.18, dd (8.0, 7.9, 1.0)	124.3
7	7.80, dd (8.1, 7.5)	135.9	7.57, dd (8.3, 7.9, 1.6)	135.2
8	7.62, d (8.1)	127.8	8.45, dd (8.3, 1.0)	122.1
8a		148.5		141.8
1′	3.44, dd (14.4, 4.8); 3.53, dd (14.4, 10.9)	35.6	4.45, dd (11.4, 4.1); 4.34, dd (11.4, 6.3)	67.5
2′		128.4	3.99, m	71.0
3′	6.93, d (8.3)	131.1	3.65, d (5.7)	64.0
4′	6.60, d (8.3)	116.5		
5′		157.5		
6′	6.60, d (8.3)	116.5		
7′	6.93, d (8.3)	131.1		
8′	5.40, dd (10.9, 4.8)	62.3		
9′		172.2		

**Table 2:** The α-glucosidase inhibitory and antioxidant activities of compounds **1**–**12**.

Compounds	α-Glucosidase Inhibitory	Antioxidant	
	IC_50_ (μM)	% Inhibition (500 μM)	IC_50_ (μM)
**1**	−	<50	−
**2**	−	<50	−
**3**	750.8	<50	−
**4**	−	<50	−
**5**	−	<50	−
**6**	−	<50	−
**7**	−	96.6	14.4
**8**	−	92	27.1
**9**	−	<50	−
**10**	−	64.9	339.3
**11**	−	<50	−
**12**	−	<50	−
Acarbose ^a^	1330		
Vitamin C ^a^		96.4	26.7

− means no activity; ^a^ positive control.

## Data Availability

All data and figures in this study are openly available.

## Supplementary Materials

The following are available online. Figures S1–S34, S35 and S36: ^1^H, ^13^C-NMR, HSQC, ^1^H-^1^H COSY, HMBC, NOESY, and HRESIMS spectra of new compounds **1** and **2**, ^1^H and ^13^C-NMR spectra of compounds **3**–**12**, and computational details for ECD calculations of **1** and specific rotation calculations of **2**.

## Sample Availability

Samples of the compounds **1**–**12** are available from the authors.
