# Supplementary material for: Asperflaloids A and B from Aspergillus flavipes DZ-3, an Endophytic Fungus of Eucommia ulmoides Oliver
Source: Molecules. 2021 Jun 9;26(12):3514. doi: 10.3390/molecules26123514 (PMC8228597; doi:10.3390/molecules26123514)
Supplement: Supplementary file 1 [file molecules-26-03514-s001.zip › molecules-1251608-supplementary.pdf]

Supplementary Materials

Asperflaloids A and B from *Aspergillus flavipes* DZ-3, an Endophytic Fungus of *Eucommia ulmoides* Oliver

Wan Liu [1], Yu Liu [1], Fan Yang [1], Shouye Han [1], Jia Zhang [1], Hui Yang [1,*], Zhongbin Cheng [1,2,*] and Qin Li [1,2,*]

[1] School of Pharmacy, Henan University, Kaifeng 475004, China; 18737806806@163.com (W.L.); liuyu5230710@163.com (Y.L.); Y18992588130@126.com (F.Y.); hanshouye123@163.com (S.H.); z919395@126.com (J.Z.);

[2] Eucommia Ulmoides Cultivation and Utilization of Henan Engineering Laboratory, Kaifeng 475004, China

* Correspondence: 10200097@vip.henu.edu.cn (H.Y.); chengzhongbin@henu.edu.cn (Z.C.); liqin@henu.edu.cn (Q.L.); Tel.: +86-371-2388-3849 (Q.L.)

	Table of Contents	Page
**Figure S1**	^{1}H NMR Spectrum of **1** in Methanol-d_4 (400 MHz)	1
**Figure S2**	^{13}C NMR Spectrum of **1** in Methanol-d_4 (100 MHz)	1
**Figure S3**	HSQC Spectrum of **1** in Methanol-d_4	2
**Figure S4**	^{1}H-^{1}H COSY Spectrum of **1** in Methanol-d_4	2
**Figure S5**	HMBC Spectrum of **1** in Methanol-d_4	3
**Figure S6**	NOESY Spectrum of **1** in Methanol-d_4	3
**Figure S7**	^{1}H NMR Spectrum of **2** in Methanol-d_4 (400 MHz)	4
**Figure S8**	^{13}C NMR Spectrum of **2** in Methanol-d_4 (100 MHz)	4
**Figure S9**	HSQC Spectrum of **2** in Methanol-d_4	5
**Figure S10**	^{1}H-^{1}H COSY Spectrum of **2** in Methanol-d_4	5
**Figure S11**	HMBC Spectrum of **2** in Methanol-d_4	6
**Figure S12**	NOESY Spectrum of **2** in Methanol-d_4	6
**Figure S13**	^{1}H NMR Spectrum of **3** in DMSO-d_6 (400 MHz)	7
**Figure S14**	^{13}C NMR Spectrum of **3** in DMSO-d_6 (100 MHz)	7
**Figure S15**	^{1}H NMR Spectrum of **4** in Methanol-d_4 (400 MHz)	8
**Figure S16**	^{13}C NMR Spectrum of **4** in Methanol-d_4 (100 MHz)	8
**Figure S17**	^{1}H NMR Spectrum of **5** in Methanol-d_4 (400 MHz)	9
**Figure S18**	^{13}C NMR Spectrum of **5** in Methanol-d_4 (100 MHz)	9

**Figure S19**	^{1}H NMR Spectrum of **6** in Methanol-d_4 (400 MHz)	10
**Figure S20**	^{13}C NMR Spectrum of **6** in Methanol-d_4 (100 MHz)	10
**Figure S21**	^{1}H NMR Spectrum of **7** in Methanol-d_4 (400 MHz)	11
**Figure S22**	^{13}C NMR Spectrum of **7** in Methanol-d_4 (100 MHz)	11
**Figure S23**	^{1}H NMR Spectrum of **8** in Methanol-d_4 (400 MHz)	12
**Figure S24**	^{13}C NMR Spectrum of **8** in Methanol-d_4 (100 MHz)	12
**Figure S25**	^{1}H NMR Spectrum of **9** in Methanol-d_4 (400 MHz)	13
**Figure S26**	^{13}C NMR Spectrum of **9** in Methanol-d_4 (100 MHz)	13
**Figure S27**	^{1}H NMR Spectrum of **10** in Methanol-d_4 (400 MHz)	14
**Figure S28**	^{13}C NMR Spectrum of **10** in Methanol-d_4 (100 MHz)	14
**Figure S29**	^{1}H NMR Spectrum of **11** in Methanol-d_4 (400 MHz)	15
**Figure S30**	^{13}C NMR Spectrum of **11** in Methanol-d_4 (100 MHz)	15
**Figure S31**	^{1}H NMR Spectrum of **12** in Methanol-d_4 (400 MHz)	16
**Figure S32**	^{13}C NMR Spectrum of **12** in Methanol-d_4 (100 MHz)	16
**Figure S33**	HRESIMS Spectrum of **1**	17
**Figure S34**	HRESIMS Spectrum of **2**	17
**S35**	Details for ECD calculations of **1**	18
**S36**	Details for specific rotation calculations of **2**	19

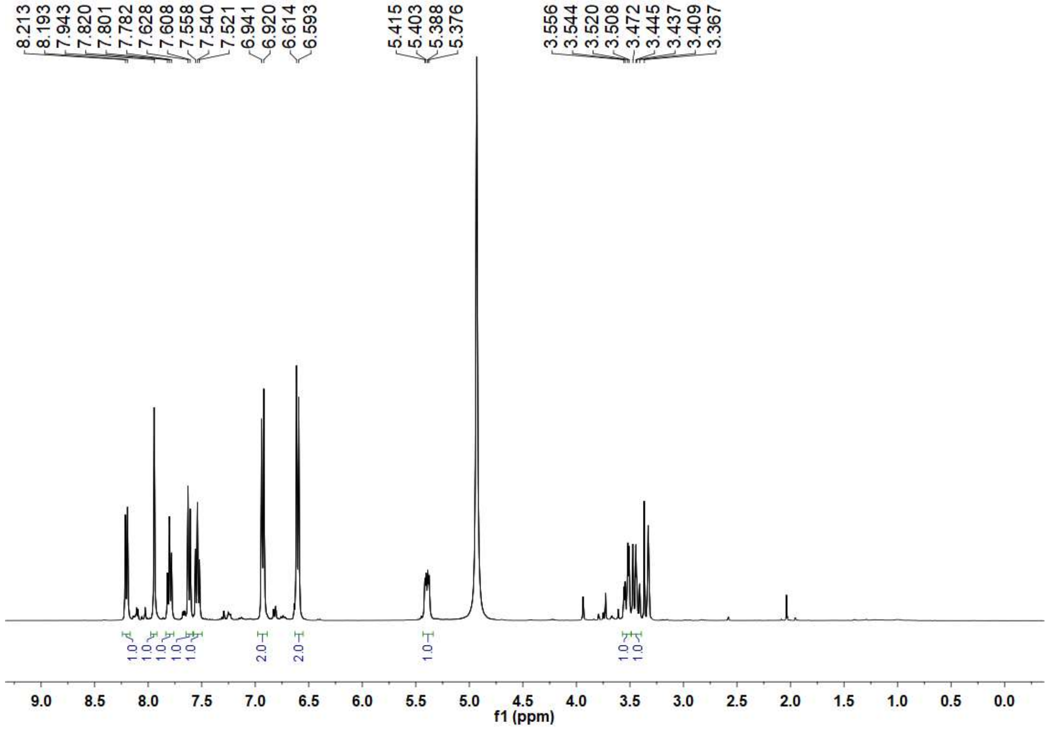

Figure S1 ^{1}H NMR Spectrum of **1** in Methanol-d_4 (400 MHz)

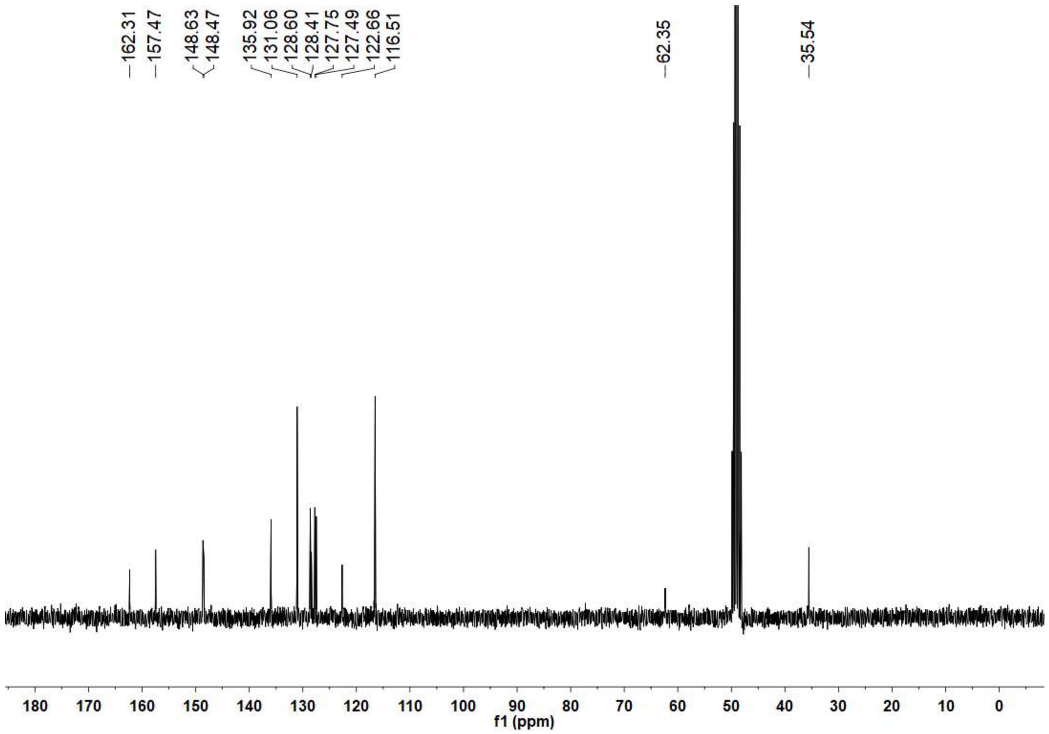

Figure S2 ^{13}C NMR Spectrum of **1** in Methanol-d_4 (100 MHz)

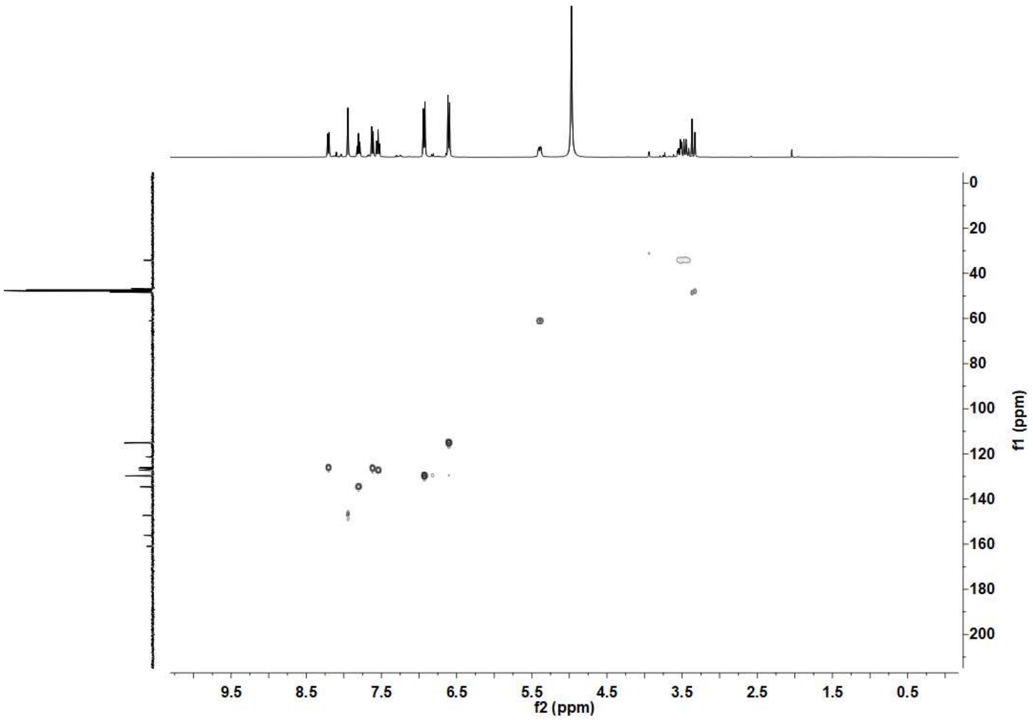

Figure S3 HSQC Spectrum of **1** in Methanol-d_4

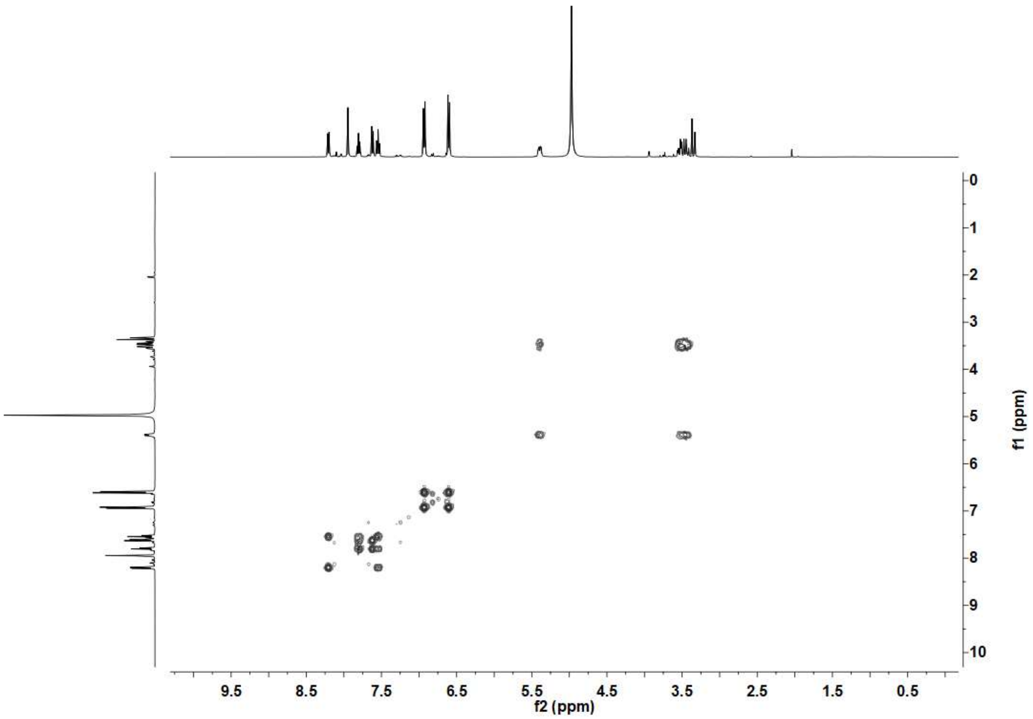

Figure S4 ^{1}H-^{1}H COSY Spectrum of **1** in Methanol-d_4

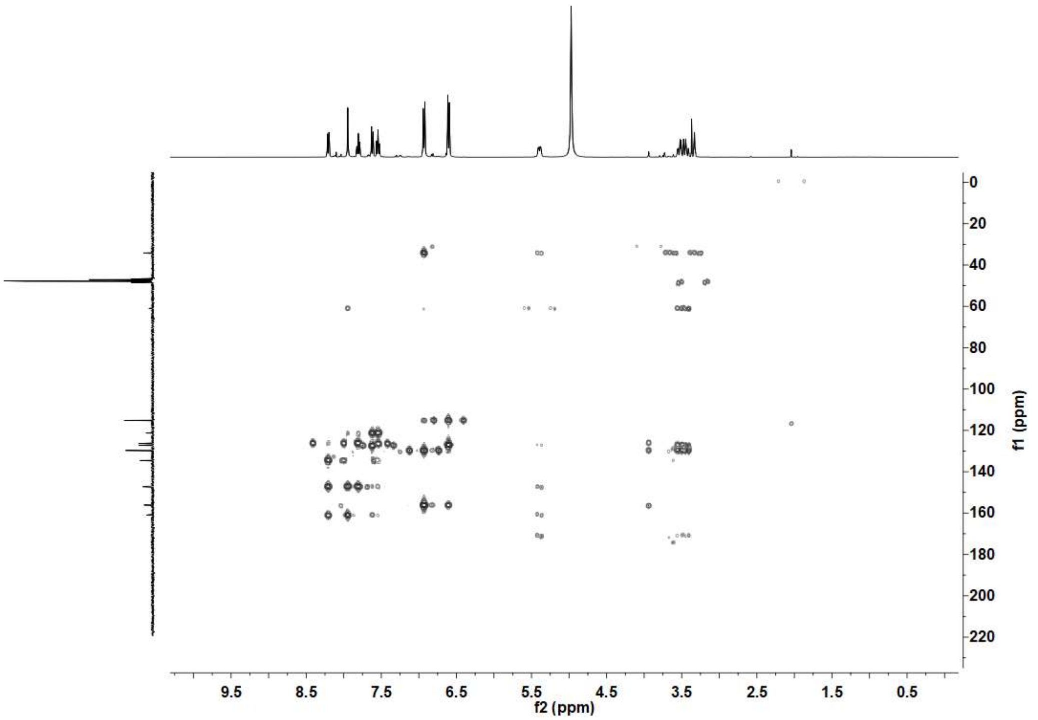

Figure S5 HMBC Spectrum of **1** in Methanol-d_4

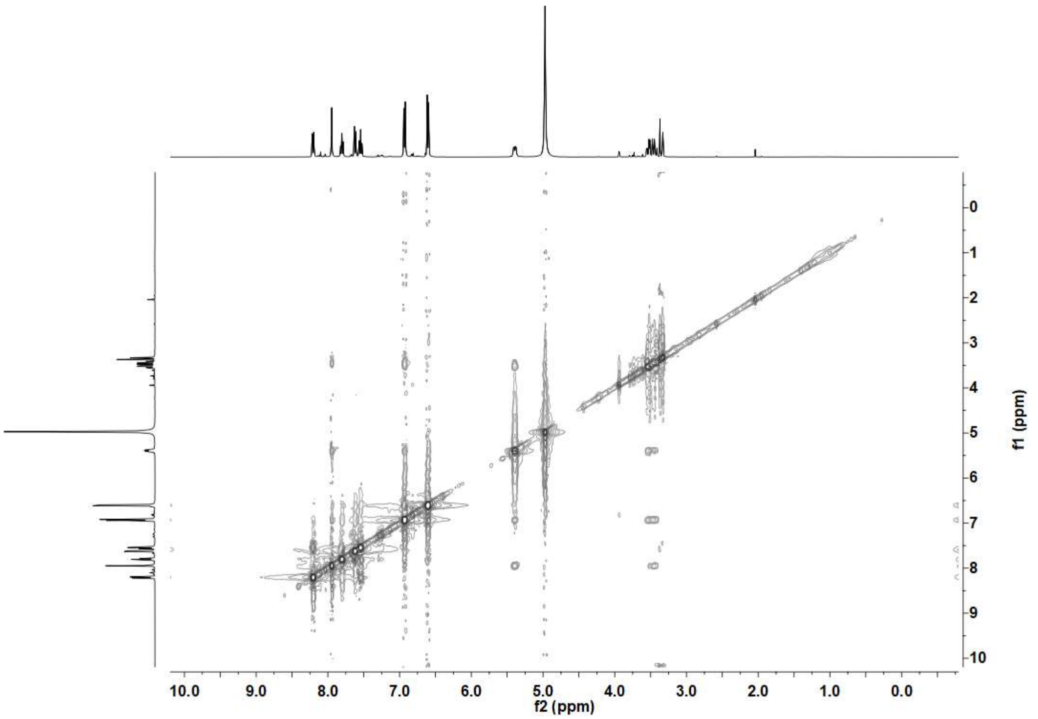

Figure S6 NOESY Spectrum of **1** in Methanol-d_4

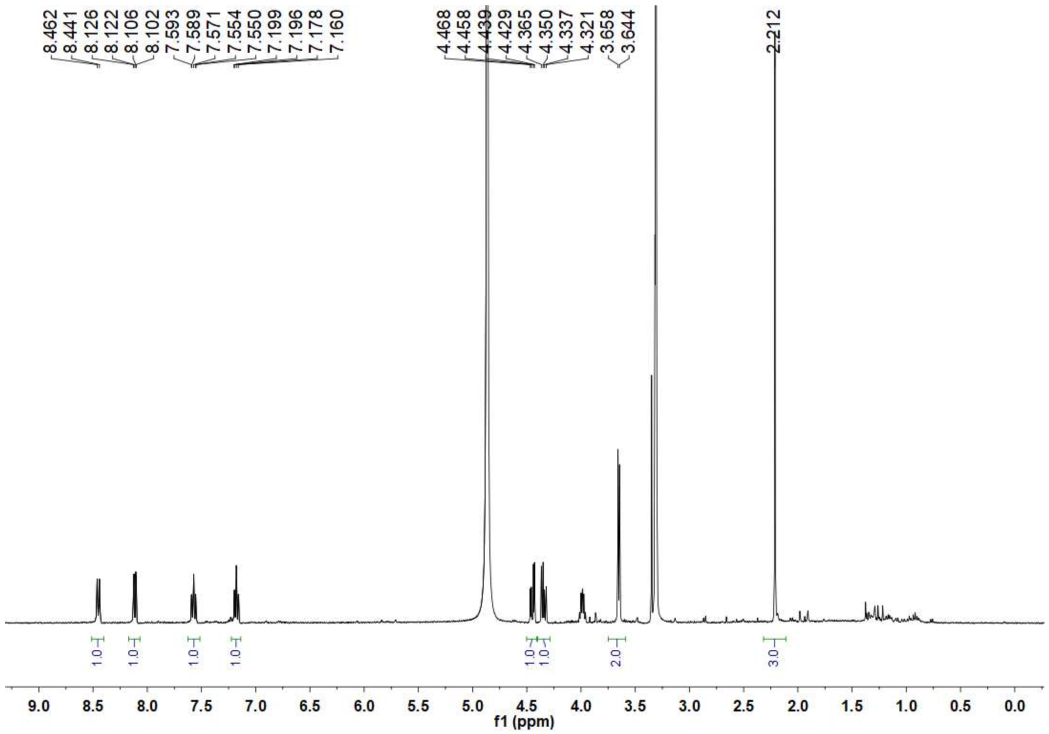

Figure S7 ^{1}H NMR Spectrum of **2** in Methanol-d_4 (400 MHz)

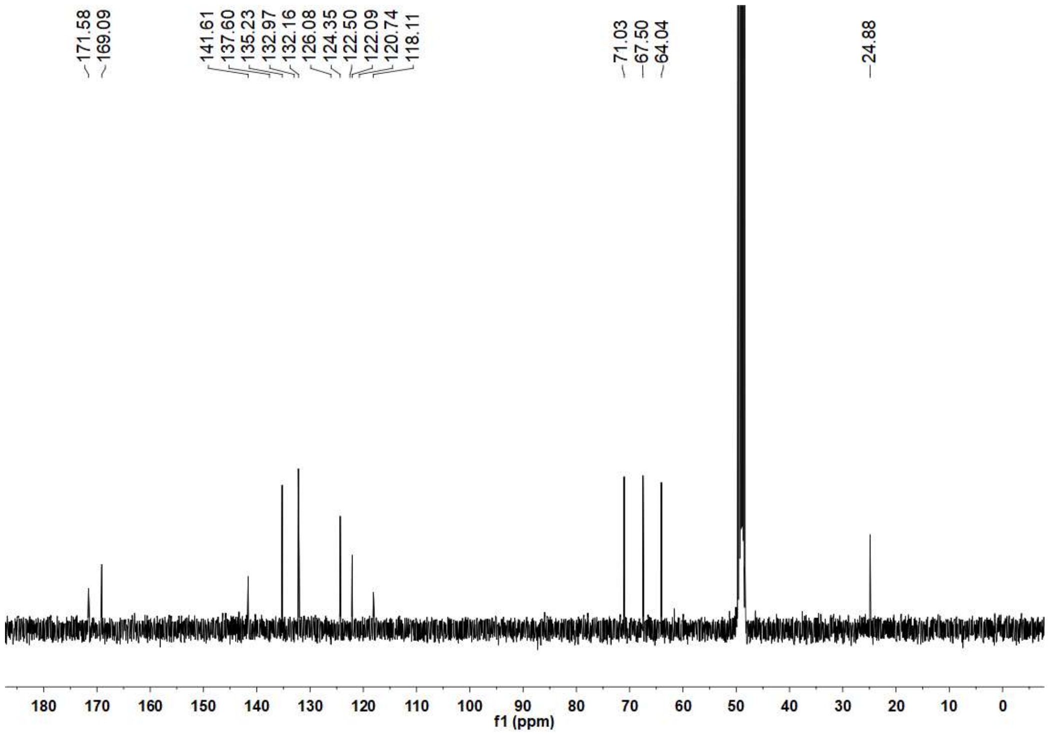

Figure S8 ^{13}C NMR Spectrum of **2** in Methanol-d_4 (100 MHz)

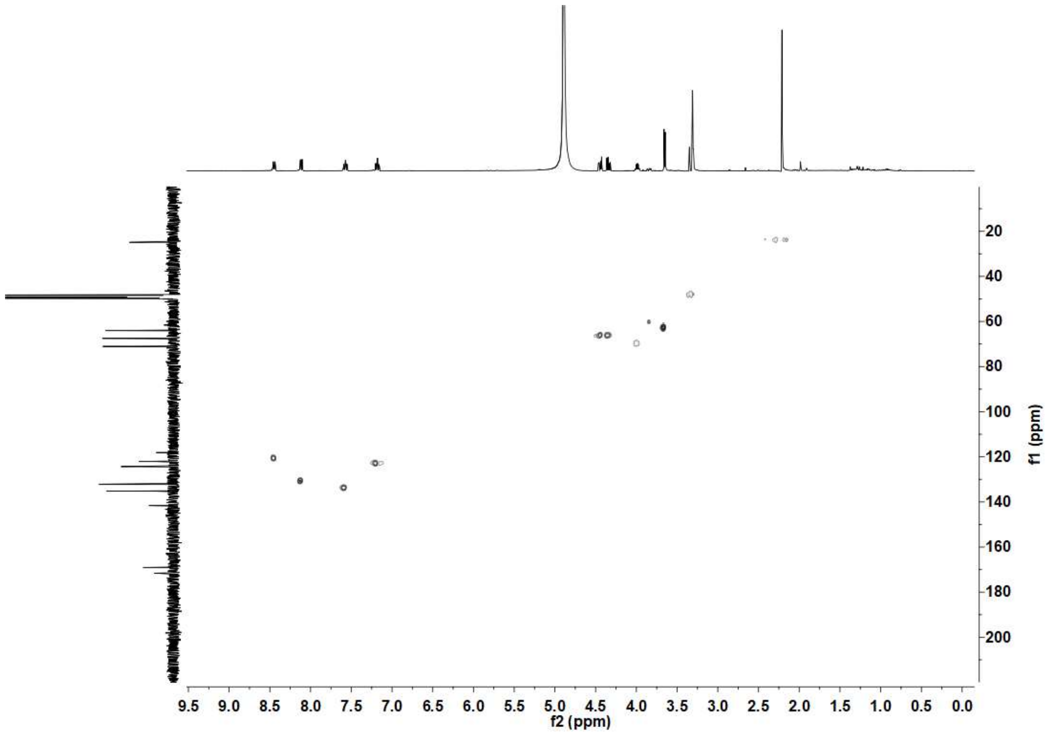

Figure S9 HSQC Spectrum of **2** in Methanol-d_4

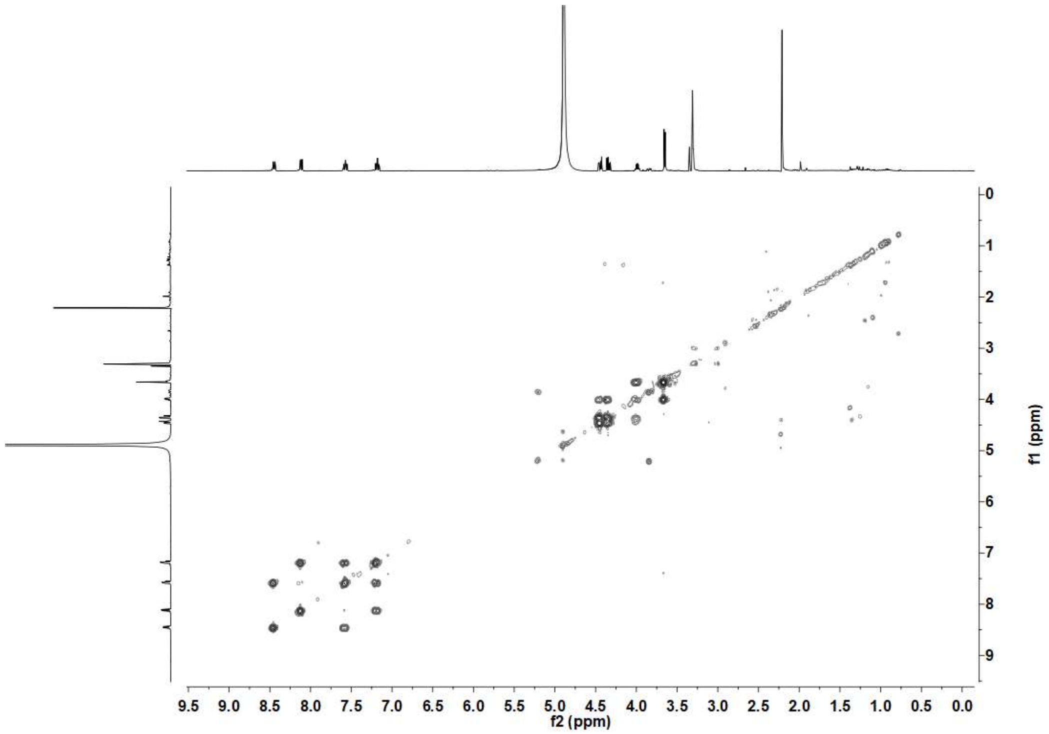

Figure S10 ^{1}H-^{1}H COSY Spectrum of **2** in Methanol-d_4

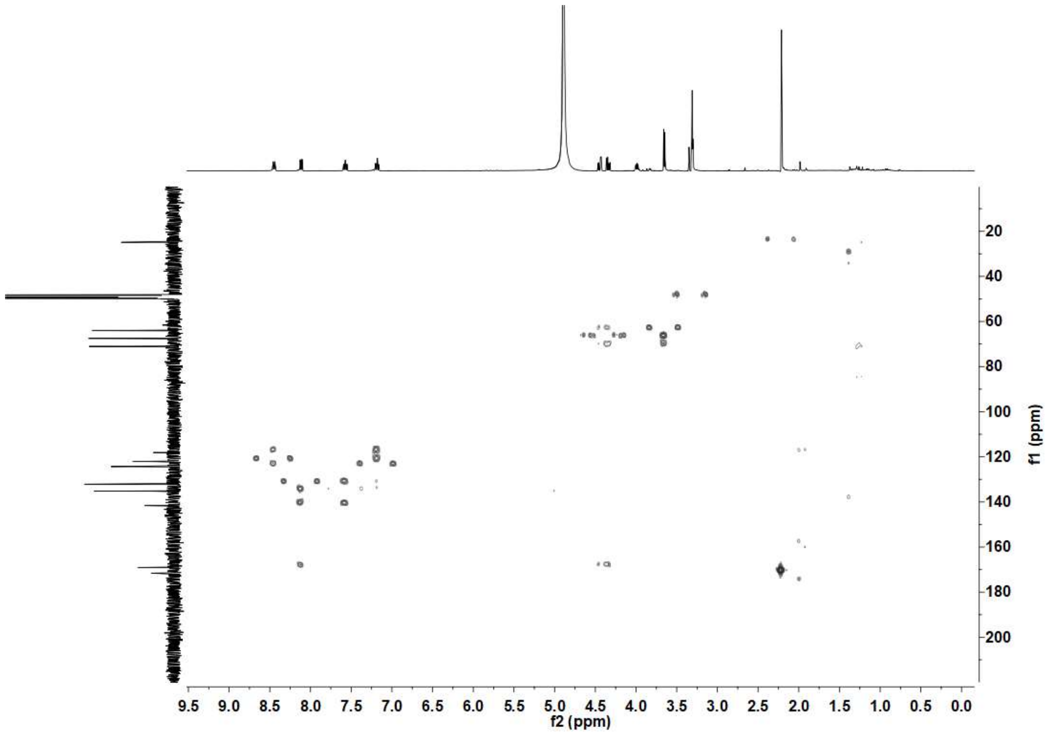

Figure S11 HMBC Spectrum of **2** in Methanol-d_4

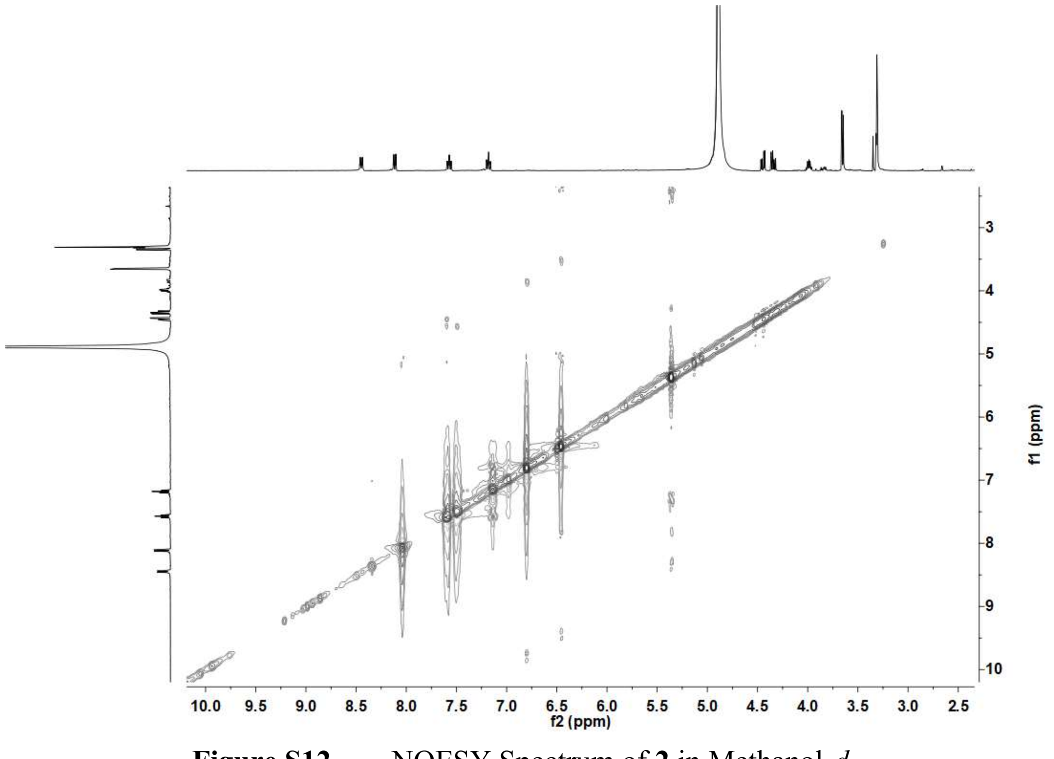

Figure S12 NOESY Spectrum of **2** in Methanol-d_4

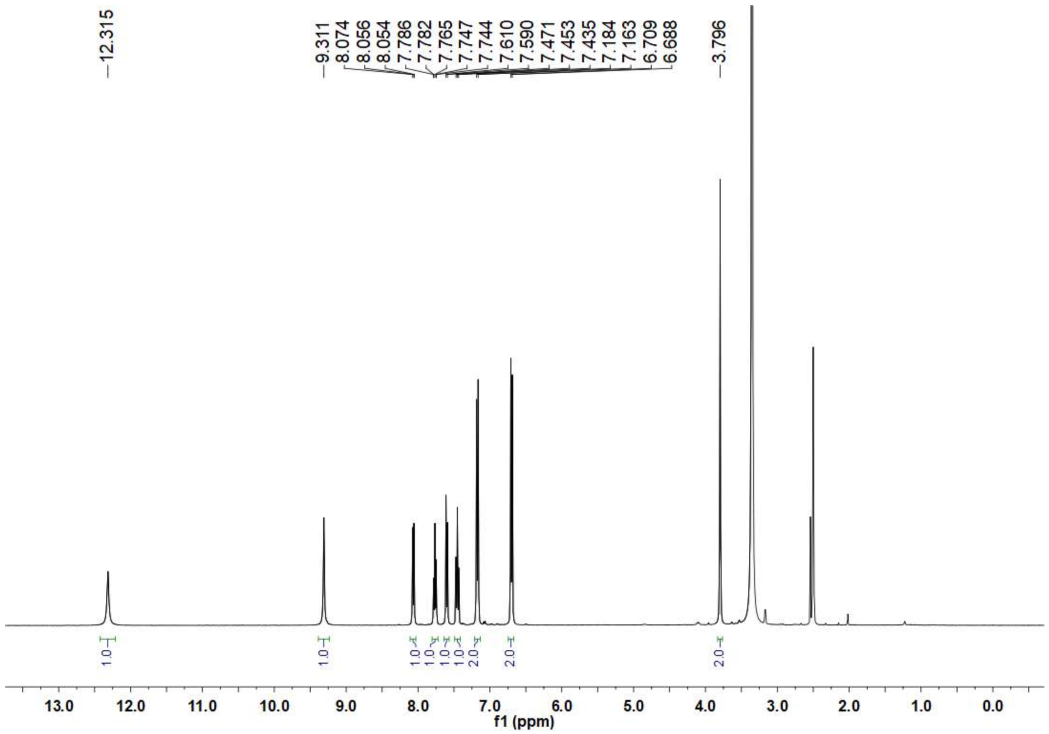

Figure S13 ^{1}H NMR Spectrum of **3** in DMSO-d_6 (400 MHz)

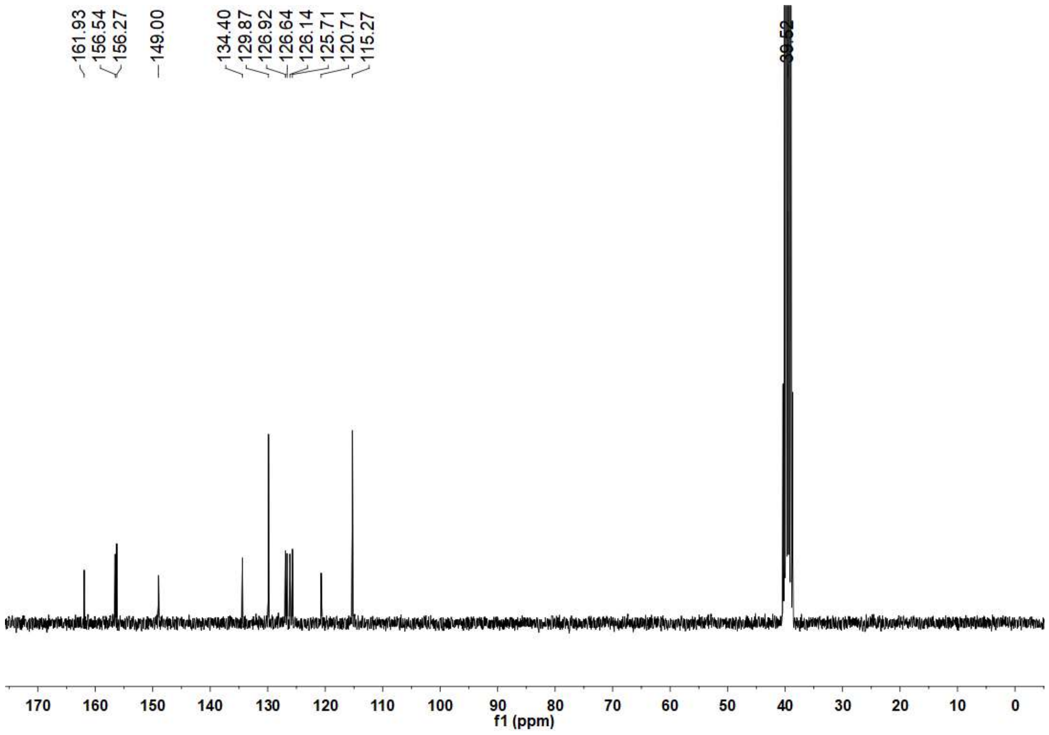

Figure S14 ^{13}C NMR Spectrum of **3** in DMSO-d_6 (100 MHz)

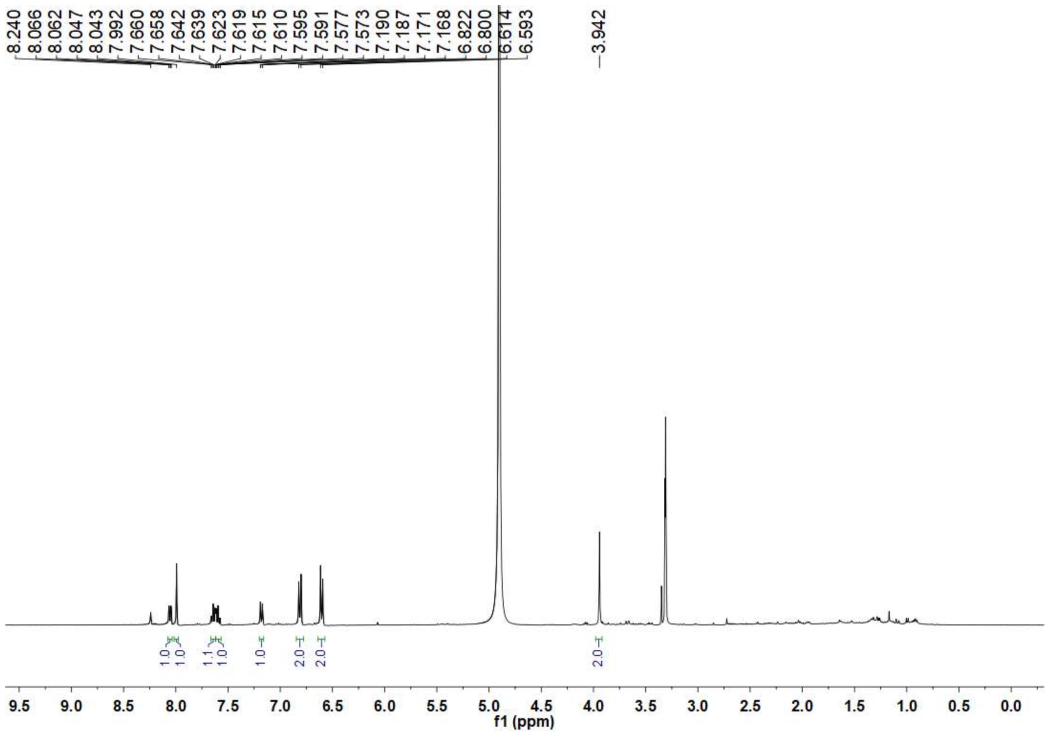

Figure S15 ^{1}H NMR Spectrum of **4** in Methanol-d_4 (400 MHz)

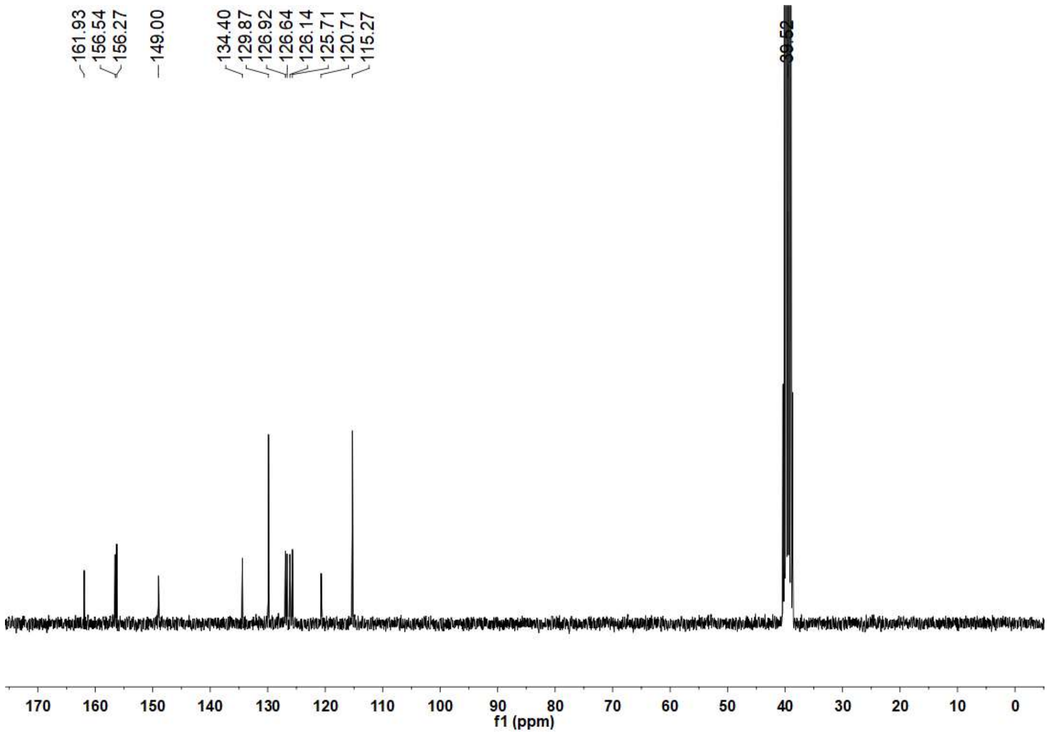

Figure S16 ^{13}C NMR Spectrum of **4** in Methanol-d_4 (100 MHz)

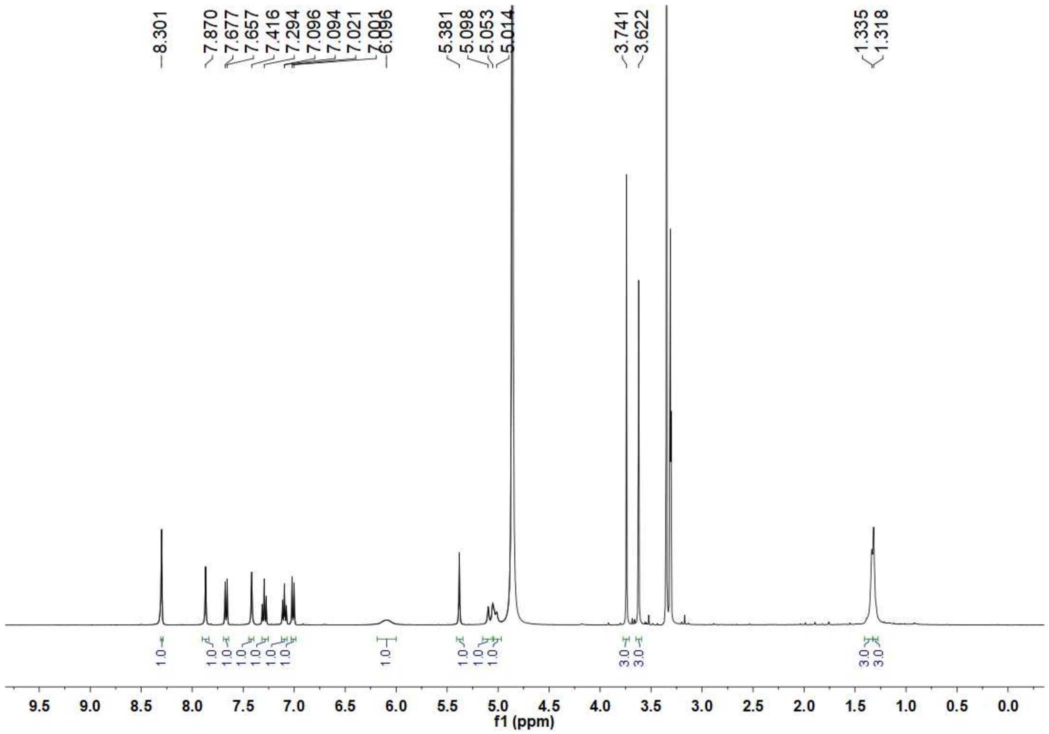

Figure S17 ^{1}H NMR Spectrum of **5** in Methanol-d_4 (400 MHz)

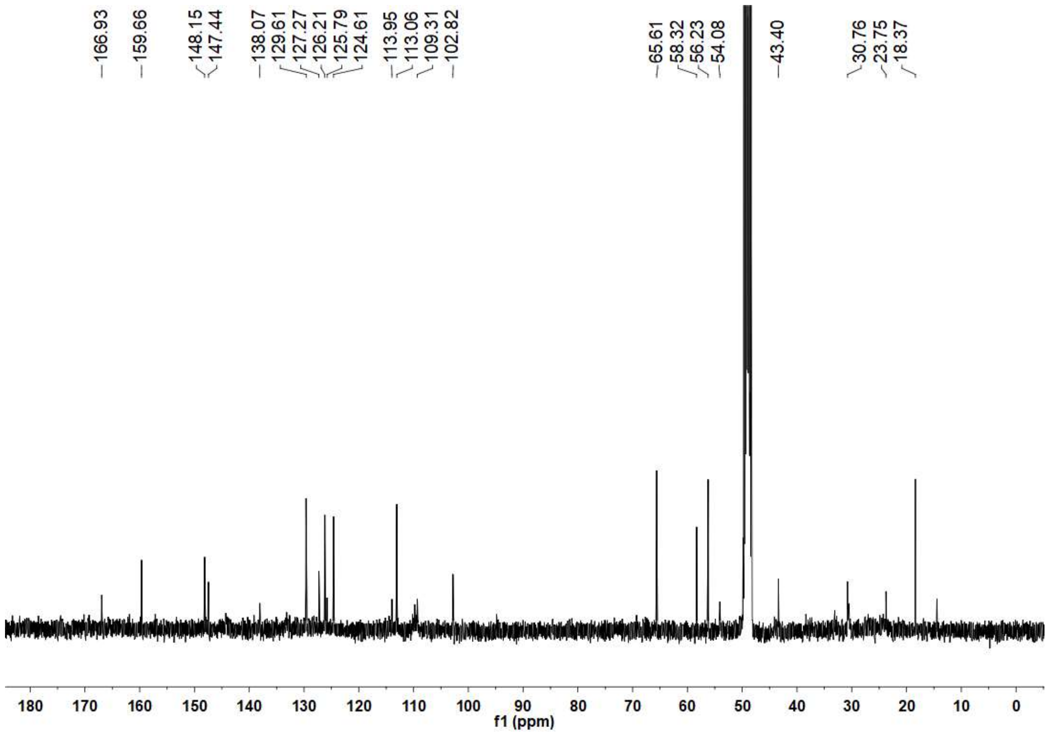

Figure S18 ^{13}C NMR Spectrum of **5** in Methanol-d_4 (100 MHz)

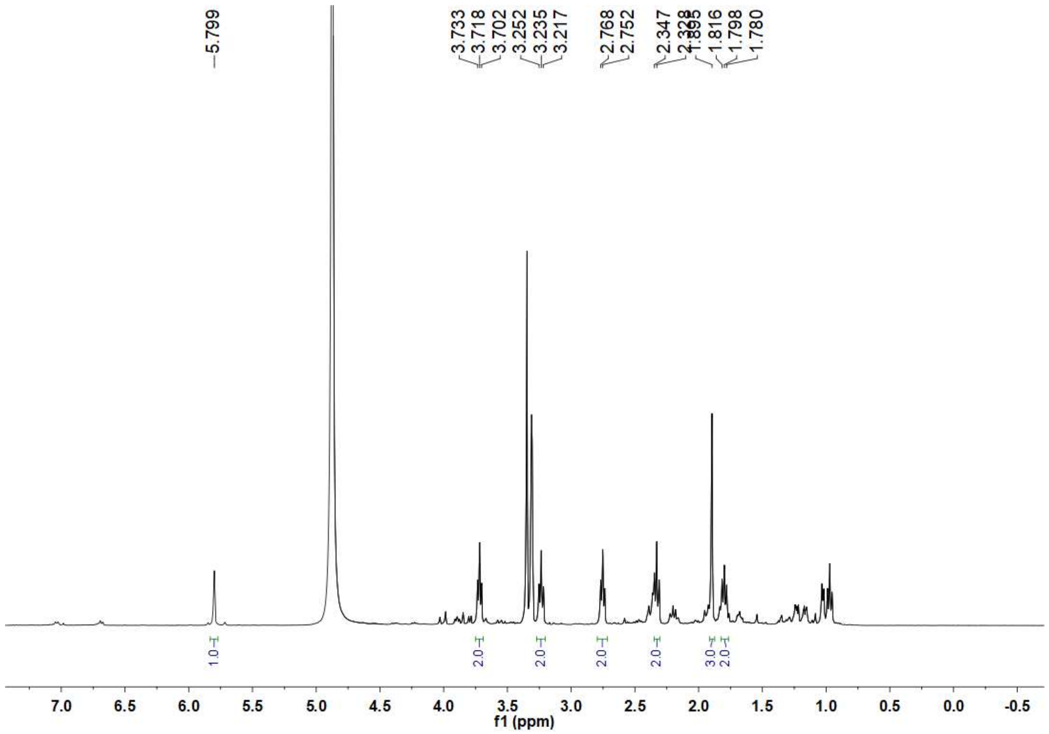

Figure S19 ^{1}H NMR Spectrum of **6** in Methanol-d_4 (400 MHz)

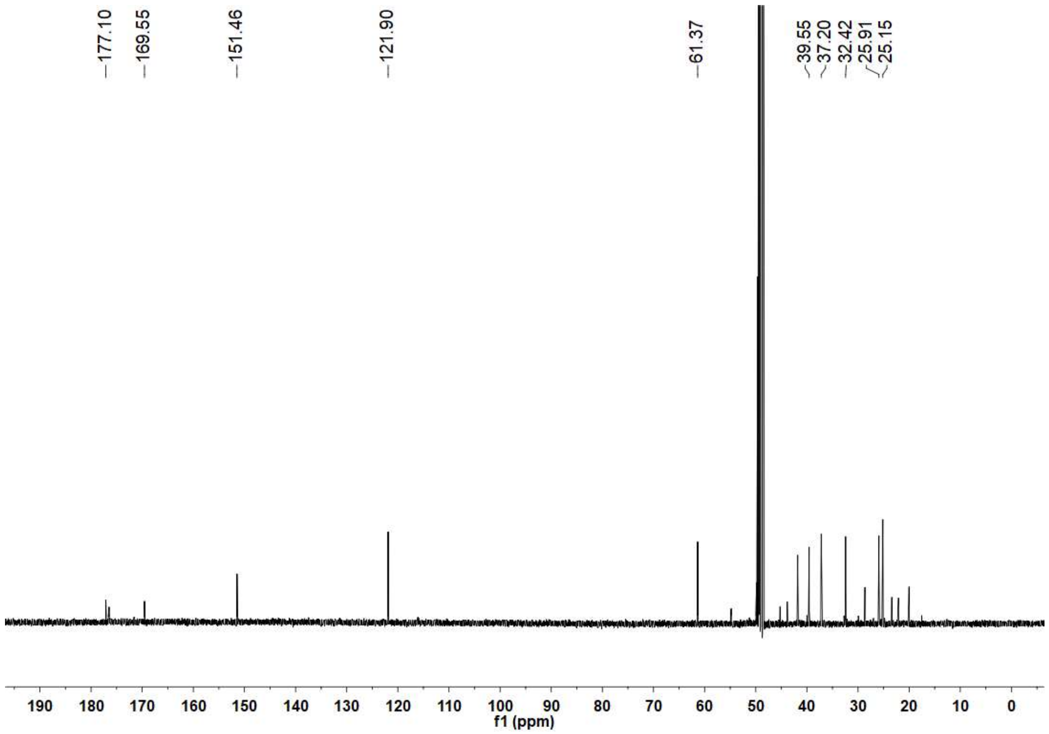

Figure S20 ^{13}C NMR Spectrum of **6** in Methanol-d_4 (100 MHz)

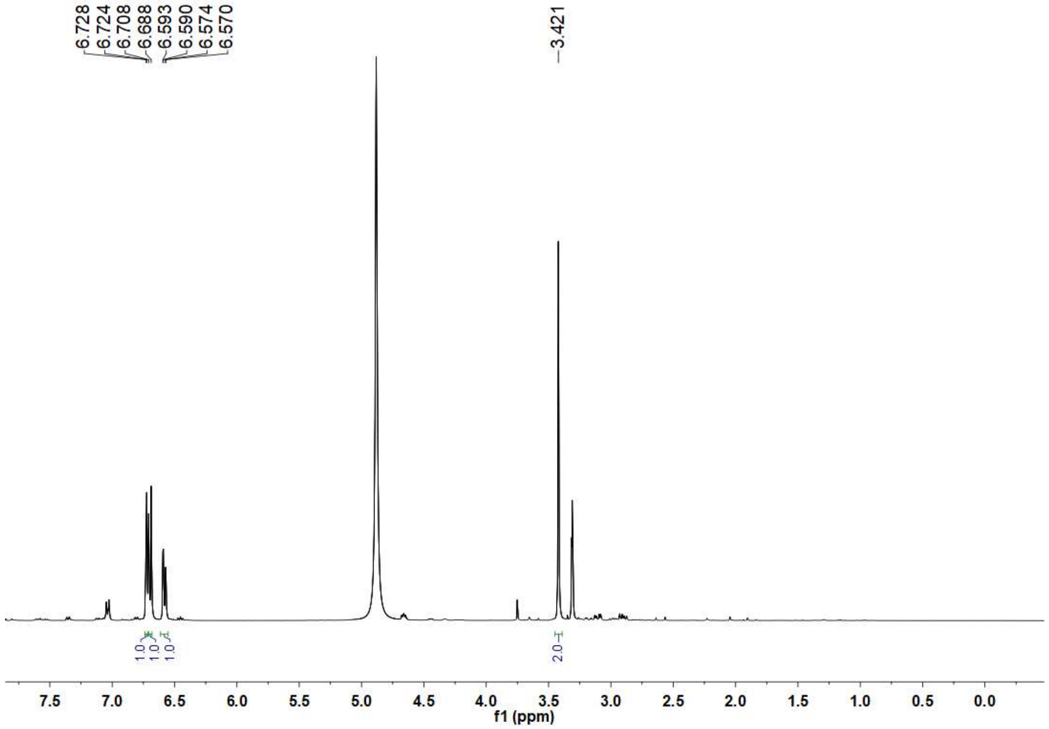

Figure S21 ^{1}H NMR Spectrum of **7** in Methanol-d_4 (400 MHz)

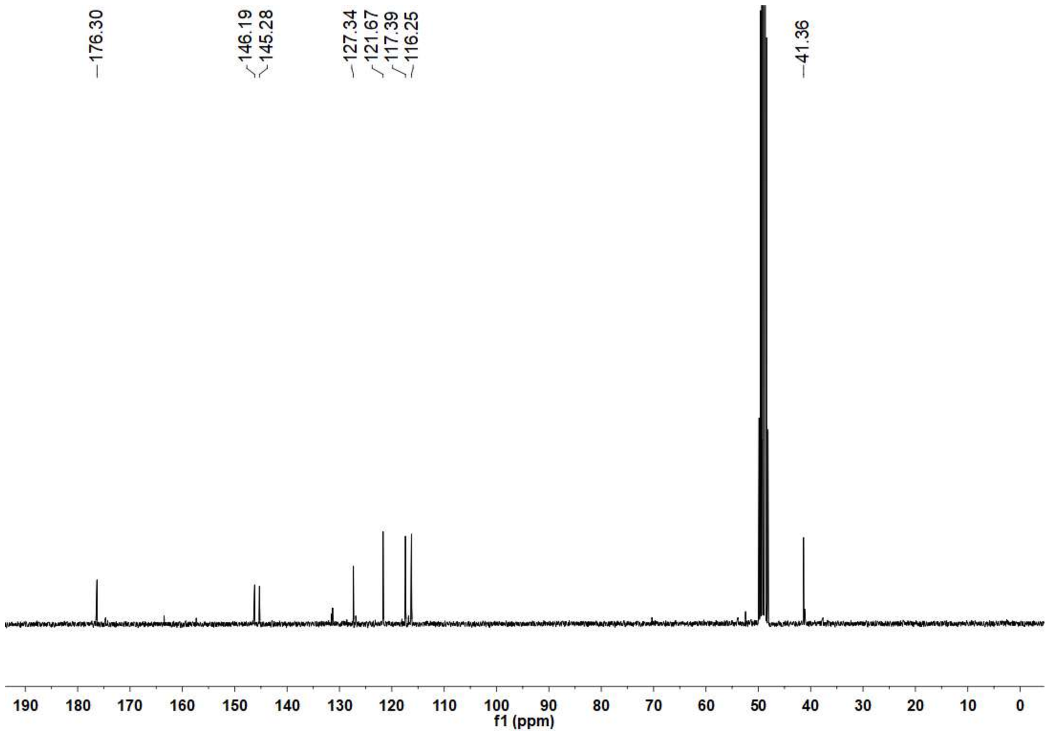

Figure S22 ^{13}C NMR Spectrum of **7** in Methanol-d_4 (100 MHz)

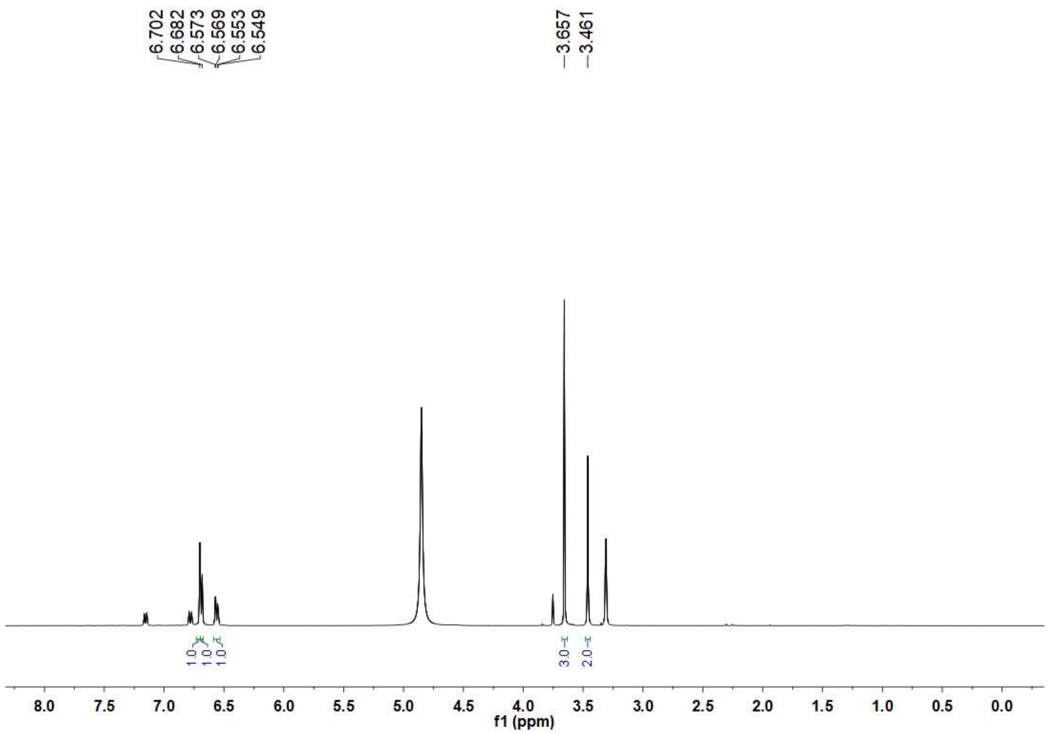

Figure S23 ^{1}H NMR Spectrum of **8** in Methanol-d_4 (400 MHz)

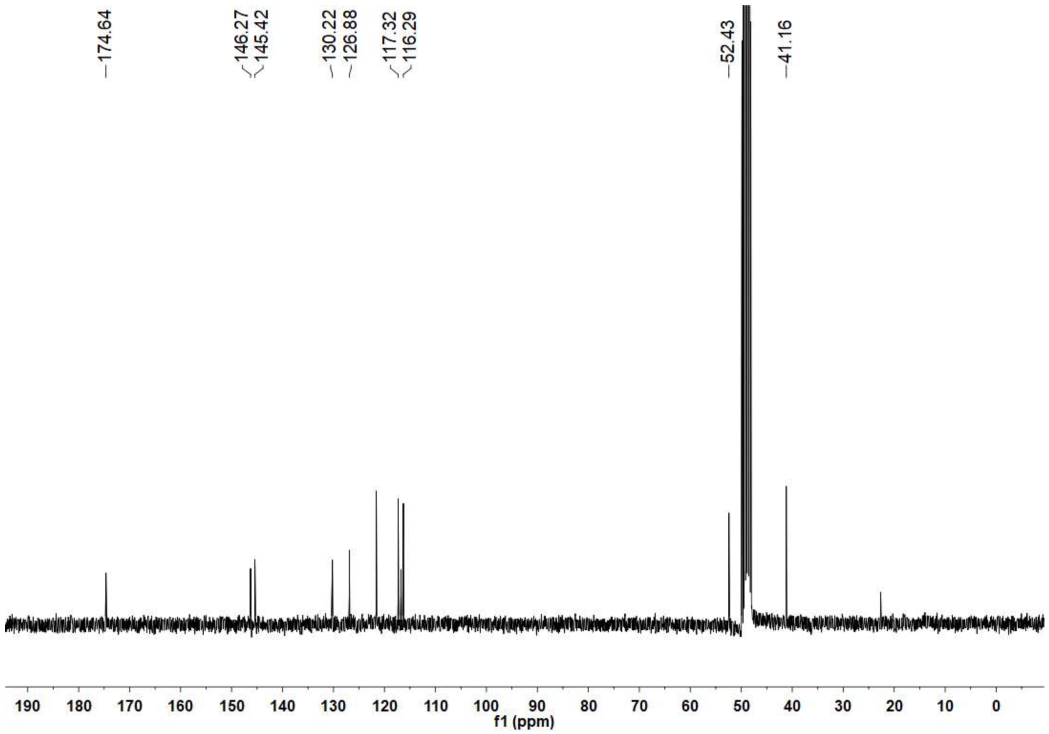

Figure S24 ^{13}C NMR Spectrum of **8** in Methanol-d_4 (100 MHz)

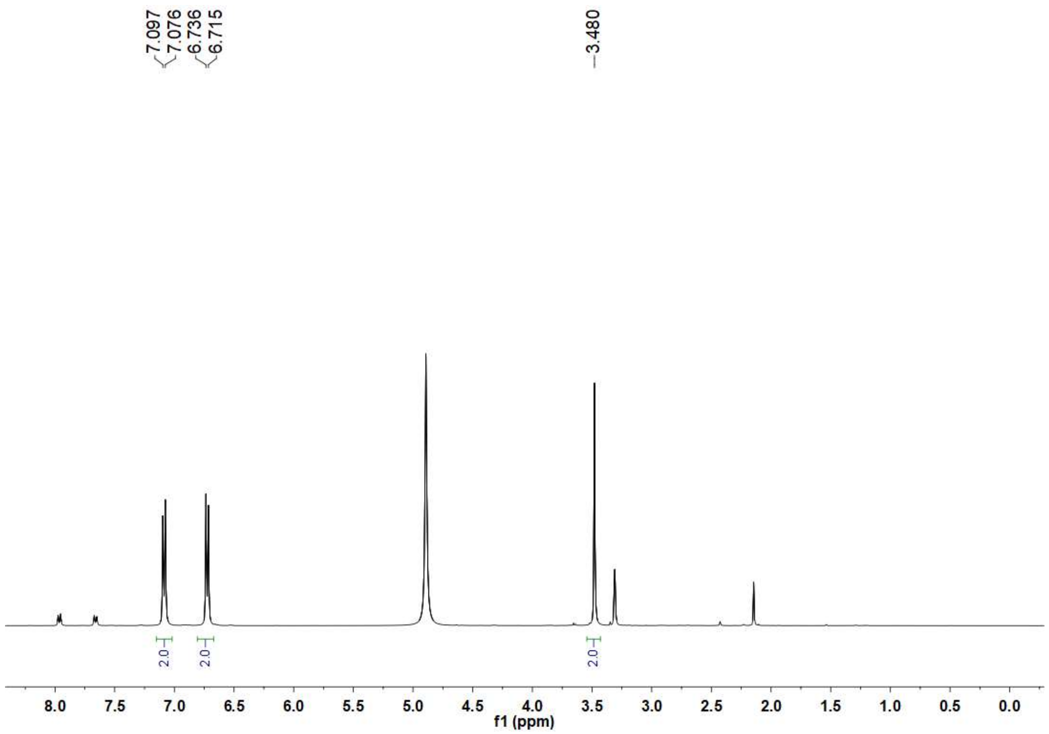

Figure S25 ^{1}H NMR Spectrum of **9** in Methanol-d_4 (400 MHz)

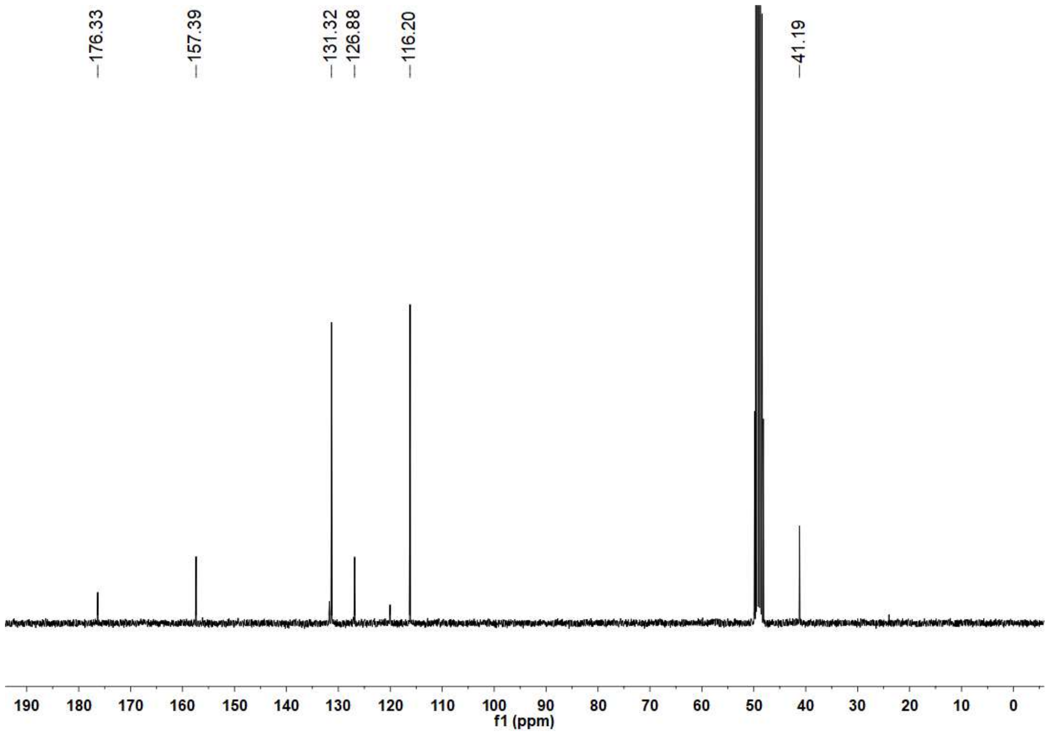

Figure S26 ^{13}C NMR Spectrum of **9** in Methanol-d_4 (100 MHz)

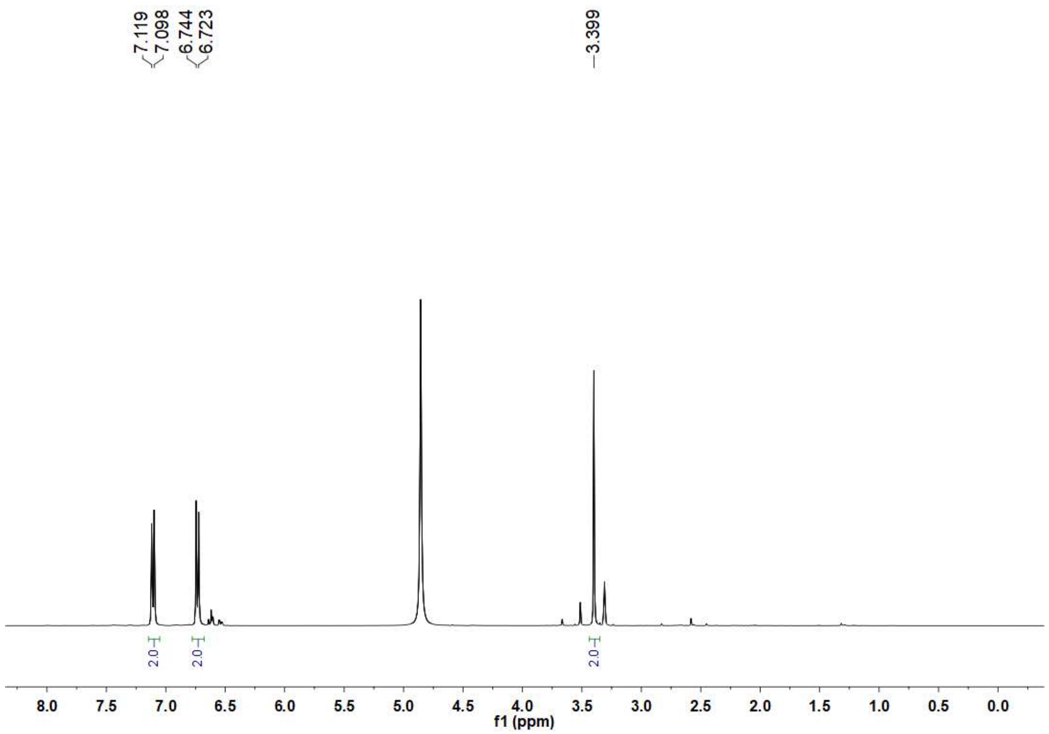

Figure S27 ^{1}H NMR Spectrum of **10** in Methanol-d_4 (400 MHz)

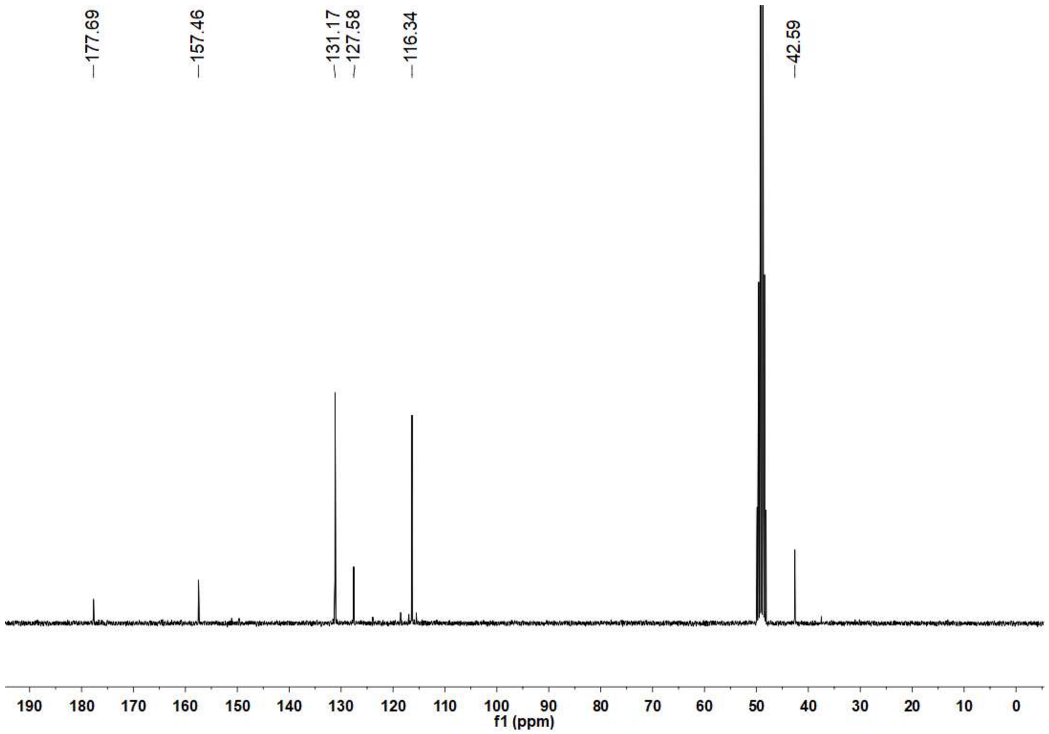

Figure S28 ^{13}C NMR Spectrum of **10** in Methanol-d_4 (100 MHz)

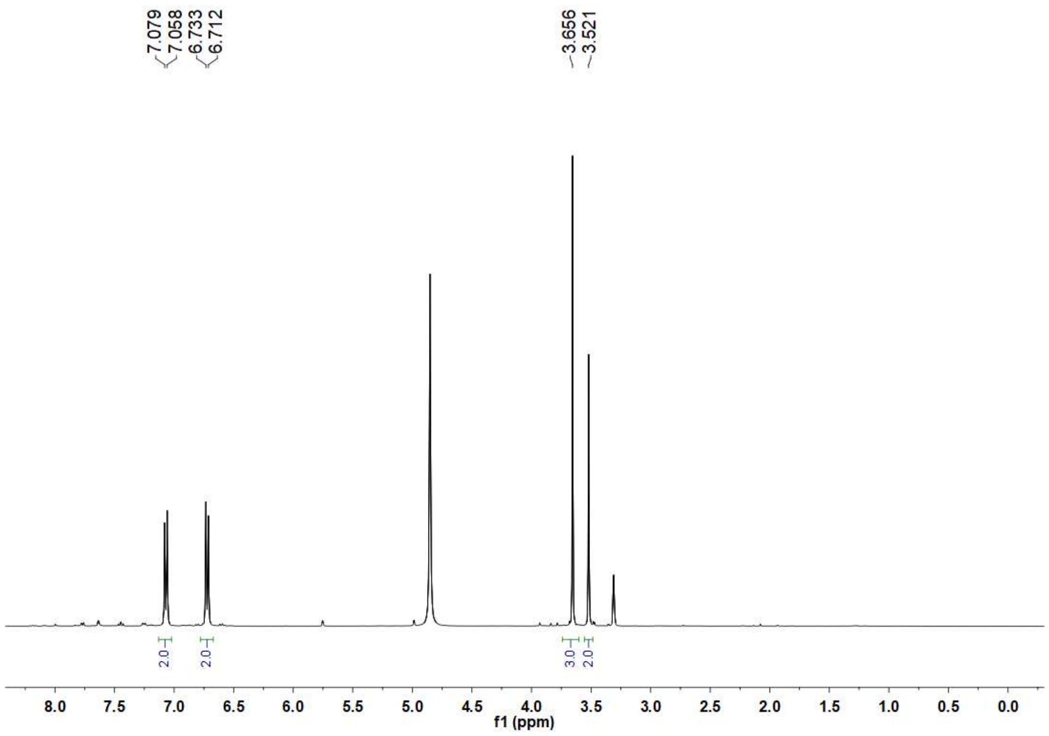

Figure S29 ^{1}H NMR Spectrum of **11** in Methanol-d_4 (400 MHz)

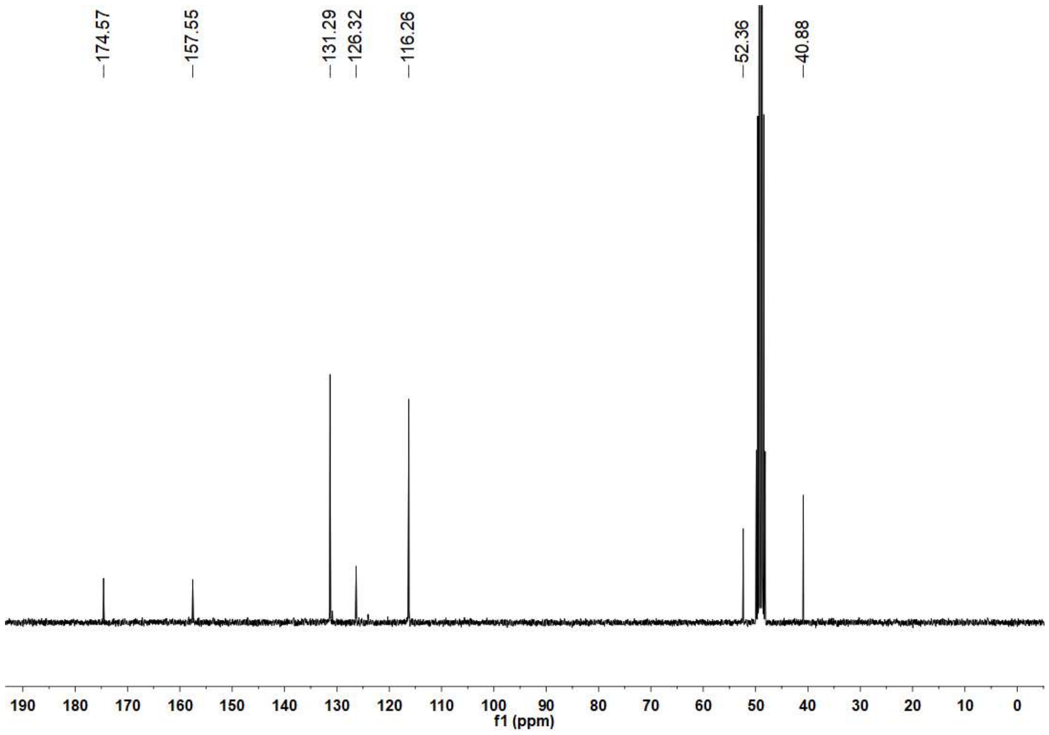

Figure S30 ^{13}C NMR Spectrum of **11** in Methanol-d_4 (100 MHz)

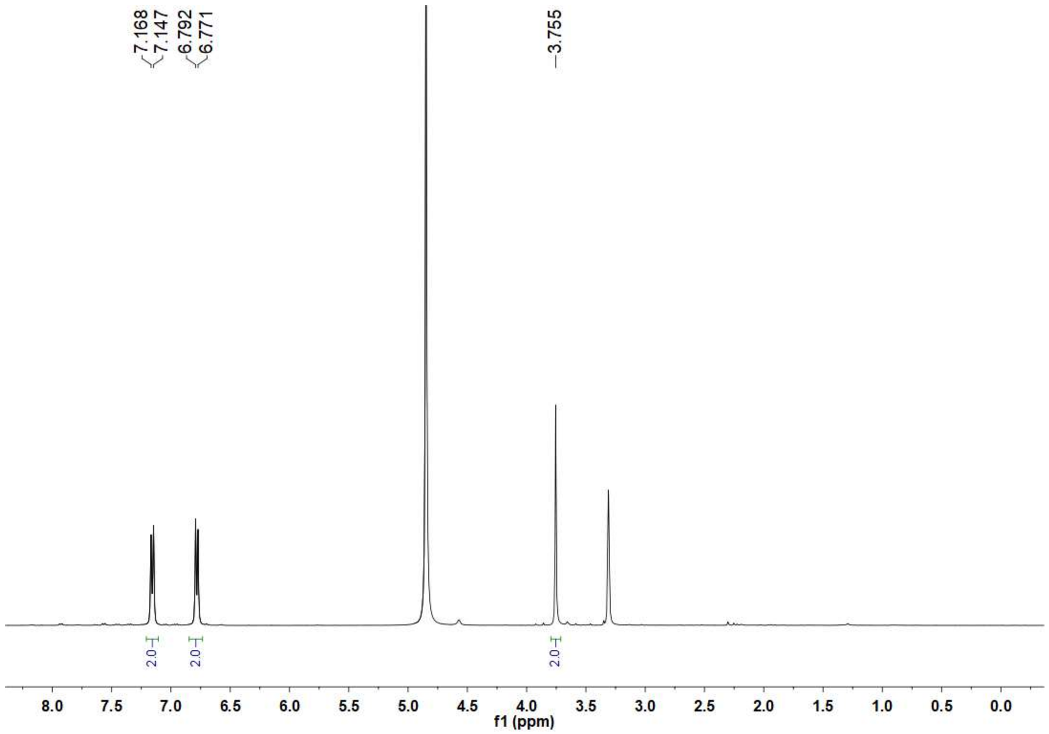

Figure S31 ^{1}H NMR Spectrum of **12** in Methanol-d_4 (400 MHz)

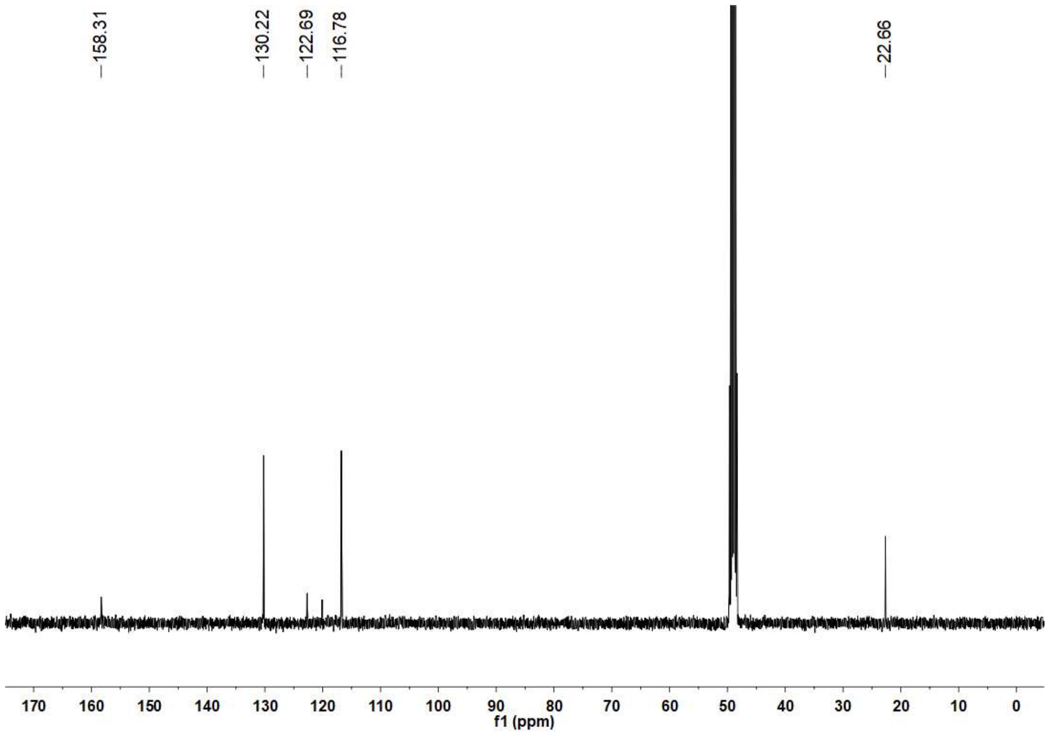

Figure S32 ^{13}C NMR Spectrum of **12** in Methanol-d_4 (100 MHz)

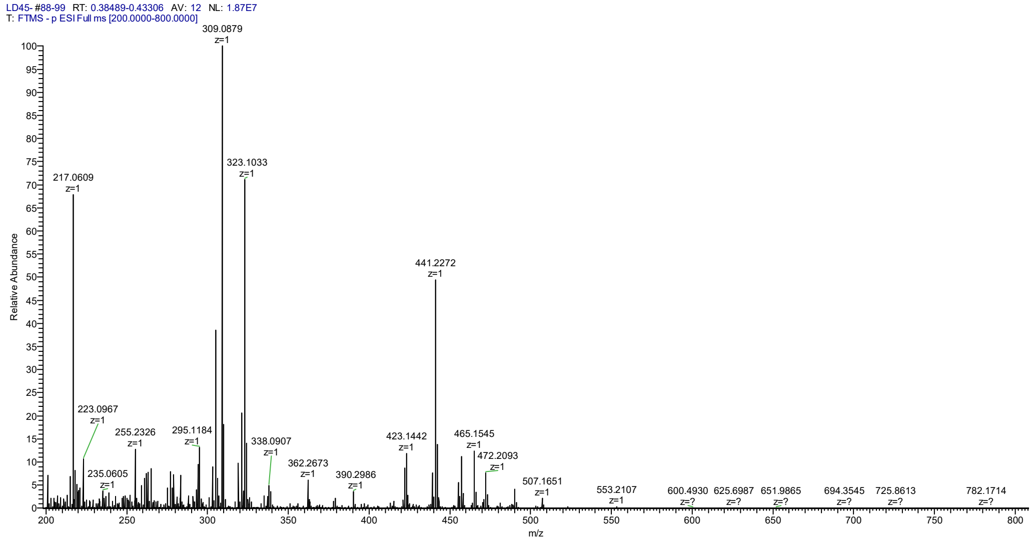

Figure S33 HRESIMS spectrum of **1**

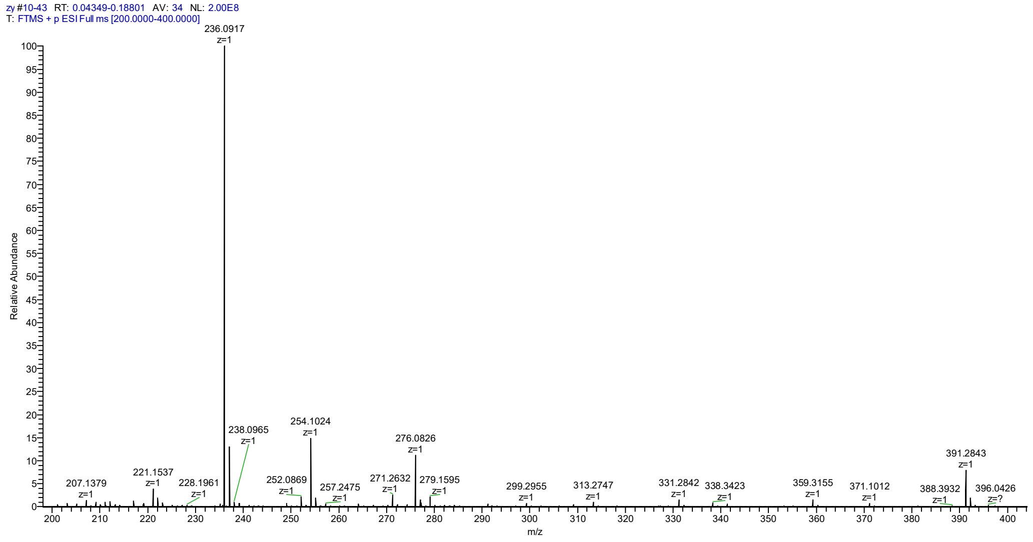

Figure S34 HRESIMS spectrum of **2**

S35 Details for ECD calculations of **1**

In general, conformational analyses were carried out via random searching in the Sybyl-X 2.0 using the MMFF94S force field with an energy cutoff of 2.5 kcal/mol.[1] The results showed 3 lowest energy conformers for (*R*)-**1.** Subsequently, the conformers were re-optimized using DFT at the b3lyp/6-31+g(d,p) level in methanol by the GAUSSIAN 09 program.[2] The energies, oscillator strengths, and rotational strengths (velocity) of the first 30 electronic excitations were calculated using the TDDFT methodology at the b3lyp/6-31+g(d,p) level in methanol. The ECD spectra were simulated by the overlapping Gaussian function (half the bandwidth at 1/e peak height, σ = 0.3, UV correction = -5 nm).[3] To get the final spectra, the simulated spectra of the conformers were averaged according to the Boltzmann distribution theory and their relative Gibbs free energy (ΔG), theoretical ECD spectrum of the corresponding enantiomer *S*-**1** was obtained by directly inverse of the ECD spectrum of *R*-**1**, respectively. By comparing the experiment spectrum with the calculated ECD spectra, the absolute configuration of the chiral center C-8' in **1** was resolved to be *S*.

Table A. Energy analysis and populations for conformers of (*R*)-**1**

conformer	Gibbs free energy (298.15 K)		
	G (Hartree)	ΔE (kcal/mol)	Population (%)
C1	-1066.768264	0	42.59
C2	-1066.768264	0	42.59
C3	-1066.7672689	0.0009951	14.82

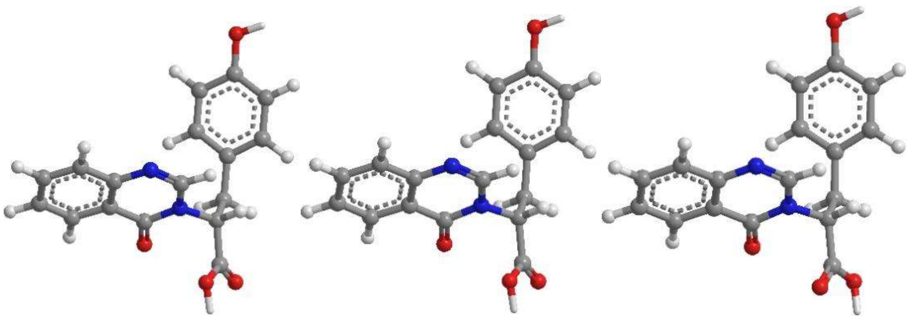

C1 C2 C3

Figure A. B3LYP-SCRF (PCM, methanol)/6-31G(d) optimized lowest energy conformers for (*R*)-**1**

S36 Details for specific rotation calculations of **2**

Conformational analyses were carried out via random searching in the Sybyl-X 2.0 using the MMFF94S force field with an energy cutoff of 2.0 kcal/mol.[1] The results showed 3 lowest energy conformers for (*R*)-**2**. The conformers were re-optimized using DFT at the b3lyp/6-31+g(d,p) level in methanol by the GAUSSIAN 09 program (Table B and Figure B).[2] The specific rotations for each conformer were calculated using the TDDFT methodology at the b3lyp/6-31+g(d) level in methanol. The specific rotations obtained for the conformers were averaged according to the Boltzmann distribution theory and their relative Gibbs free energy (ΔG) to give the specific rotation of (*R*)-**2** (Table B), the specific rotation of (*S*)-**2** was theoretically determined by taking the opposite value of (*R*)-**2**. By comparing the experiment data ($[\alpha]^{20}_{D}$ +270) with the calculated data (*R*-**2**:$[\alpha]^{20}_{D}$ -147; *S*-**2**: $[\alpha]^{20}_{D}$ +147), the absolute configuration of the chiral center C-2' in **2** was resolved to be *S*.

Table B. Energy analysis for conformers of (*R*)-**2** and the calculated specific rotations.

conformer	Gibbs free energy (298.15 K)			Specific rotations	Averaged
	G (Hartree)	ΔE (kcal/mol)	Population (%)		
C1	-897.304147	0	52.79	-151.53	
C2	-897.303654	0.000493	31.31	-162.42	-147.05
C3	-897.303014	0.001133	15.90	-101.90	

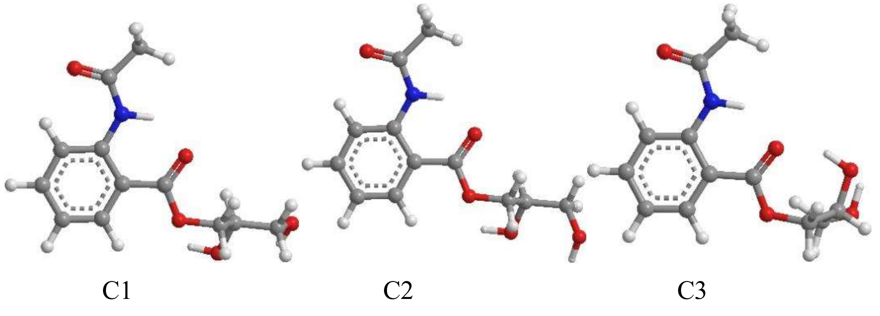

Figure B. B3LYP-SCRF (smd, methanol)/6-31G(d) optimized lowest energy conformers for (*R*)-**2**.

References

1. Sybyl Software, version X 2.0; Tripos Associates Inc.: St. Louis, MO, 2013.

2. Frisch, M. J.; Trucks, G. W.; Schlegel, H. B.; Scuseria, G. E.; Robb, M. A.; Cheeseman, J. R.; Scalmani, G.; Barone, V.; Mennucci, B.; Petersson, G. A.; Nakatsuji, H.; Caricato, M.; Li, X.; Hratchian, H. P.; Izmaylov, A. F.; Bloino, J.; Zheng, G.; Sonnenberg, J. L.; Hada, M.; Ehara, M.; Toyota, K.; Fukuda, R.; Hasegawa, J.; Ishida, M.; Nakajima, T.; Honda, Y.; Kitao, O.; Nakai, H.; Vreven, T.; Montgomery, Jr., J. A.; Peralta, J. E.; Ogliaro, F.; Bearpark, M.; Heyd, J. J.; Brothers, E.; Kudin, K. N.; Staroverov, V. N.; Kobayashi, R.; Normand, J.; Raghavachari, K.; Rendell, A.; Burant, J. C.; Iyengar, S. S.; Tomasi, J.; Cossi, M.; Rega, N.; Millam, J. M.; Klene, M.; Knox, J. E.; Cross, J. B.; Bakken, V.; Adamo, C.; Jaramillo, J.; Gomperts, R.; Stratmann, R. E.; Yazyev, O.; Austin, A. J.; Cammi, R.; Pomelli, C.; Ochterski, J. W.; Martin, R. L.; Morokuma, K.; Zakrzewski, V. G.; Voth, G. A.; Salvador, P.; Dannenberg, J. J.; Dapprich, S.; Daniels, A. D.; Farkas, Ö.; Foresman, J. B.; Ortiz, J. V.; Cioslowski, J.; Fox, D. J.Gaussian 09, Rev. C 01; Gaussian, Inc., Wallingford CT, 2009.

3. Stephens, P. J.; Harada, N. ECD cotton effect approximated by the Gaussian curve and other methods. *Chirality* **2010**, *22*, 229–233.
